# Soluble CD80 oral delivery by recombinant Lactococcus suppresses tumor growth by enhancing antitumor immunity

**DOI:** 10.1002/btm2.10533

**Published:** 2023-05-03

**Authors:** Ziqing Lin, Yanqing Tang, Zerong Chen, Simin Li, Xueyan Xu, Xufeng Hou, Zhenhui Chen, Junjie Wen, Weisen Zeng, Xiaojing Meng, Hongying Fan

**Affiliations:** ^1^ Department of Cell Biology, School of Basic Medicine Southern Medical University Guangzhou China; ^2^ Guangzhou Virotech Phamaceutical Co., Ltd Guangzhou China; ^3^ Department of Urology, Nanfang Hospital Southern Medical University Guangzhou China; ^4^ Department of Dermatology, Dermatology Hospital of Southern Medical University Southern Medical University Guangzhou China; ^5^ Department of Microbiology, Guangdong Provincial Key Laboratory of Tropical Disease Research, School of Public Health Southern Medical University Guangzhou China; ^6^ Guangzhou Weisengene Biological Technology Co., Ltd. Guangzhou China; ^7^ Department of Occupational Health and Occupational Medicine, School of Public Health Southern Medical University Guangzhou Guangdong China

**Keywords:** cholera toxin B subunit (CTB), human soluble CD80, lactic acid bacteria, tumor therapy

## Abstract

CD80 is an important co‐stimulatory molecule that participates in the immune response. Soluble CD80 can induce T cell activation and overcome PDL1‐mediated immune suppression. In this study, we aimed to construct recombinant *Lactococcus lactis* for oral delivery of the soluble CD80 (hsCD80) protein or the fusion protein containing the cholera toxin B subunit (CTB) and hsCD80 (CTB‐hsCD80) under the control of the nisin‐inducible expression system. The recombinant *L. lactis* expressed and secreted hsCD80 or CTB‐hsCD80 fusion proteins after induction by nisin in vitro and in the enteric cavity. Additionally, the CTB‐hsCD80 fusion protein showed uptake by intestinal epithelial cells, was cleaved by the furin protease, and was released as free hsCD80 protein into the blood circulation. Orally administered hsCD80 and CTB‐hsCD80 containing *L. lactis* increased the proportion of activated T cells in the spleen and intestinal epithelium, inhibited tumor growth, and prolonged the survival of tumor‐bearing mice. The hsCD80‐containing L. *lactis* showed greater therapeutic effects on primary colonic adenoma in APC^min/−^ mice and completely suppressed tumor growth. Further, recombinant CTB‐hsCD80 in *L. lactis* was more efficient than hsCD80‐containing bacteria in inhibiting the growth of xenografted colon cancer and melanoma cells. hsCD80 engineered probiotics may serve as a promising new approach for antitumor immunotherapy, especially for colorectal cancer.

## 1 INTRODUCTION

Spontaneous antitumor immunity is ordinarily repressed by the immune checkpoint pathway. Malignant tumors usually express various immune checkpoint molecules to escape immune surveillance by interacting with the checkpoint receptors expressed on immune cells, and this is the main obstacle for tumor immunotherapy.^1^ PD‐1 is a well‐known checkpoint receptor that belongs to the immunoglobulin gene superfamily. It is expressed on the surfaces of activated T cells, B cells, natural killer cells, and dendritic cells (DCs). PD‐1 ligand 1 (PDL1, also called B7‐H1 or CD274) and PDL2 (also called B7‐DC or CD273) are expressed either constitutively or in response to exposure to interferon γ (IFN‐γ) by many tumor cells.^2^ The interaction of PD‐1 with PDL1 or PDL2 results in immune tolerance by inducing T cell apoptosis, decreasing the killing capacity of effector T cells (Te), and inhibiting T cell activation.^3^ Recently, checkpoint inhibitors have been identified as promising antitumor drugs, as these can restore T cell activity by blocking the interaction between PD‐1 and PDL1 or PDL2. Humanized antibodies for PD‐1 or PDL1, such as nivolumab, pembrolizumab, or atezolizumab, have shown promising clinical efficacy for the treatment of melanoma, colon cancer, lung cancer, and several other malignant tumors.^4^, ^5^, ^6^, ^7^

However, recent clinical data indicate that only 17–28% of patients with advanced cancers showed complete or partial remission following PD‐1 or PDL1 antibody treatment. The majority of patients do not benefit from checkpoint inhibitor therapy.^4^, ^5^, ^6^, ^7^ The main reason for this is the high degree of heterogeneity of malignant tumors. One tumor cell usually expresses several inhibitory molecules and generates immune tolerance through multiple mechanisms, and different types of tumors have different immunosuppression mechanisms.^8^ Therefore, most malignant tumors require combination therapy with multiple drugs or alternative routes to achieve curative results.

Humanized antibodies are expensive for low‐ and middle‐income families, especially when continuous treatment is required. Moreover, these drugs are administered via injection, which may cause side effects such as allergy, infection, and local vascular stimulation in patients.^9^, ^10^ Therefore, it is necessary to explore new strategies to overcome immune suppression and to identify novel checkpoint inhibitors.

CD80 is an immune co‐stimulatory molecule, which plays an important role in antitumor and anti‐viral immunity.^11^ CD80, also known as B7‐1, was first cloned from active B cells, and is a type 1 transmembrane protein of the immunoglobulin superfamily. Molecules homologous to CD80 include PDL1 and PDL2.^12^ Full activation of T cells requires that the main signal is recognized by the T‐cell receptor and antigen peptide/major histocompatibility complex, and the auxiliary signals driven by co‐stimulatory molecules, such as CD80 and CD86, which are present on tumor or antigen‐presenting cells (APCs). The engagement of CD80 with its positive receptor CD28 enhances T cell proliferation and IL‐2 secretion.^13^ In contrast, CD80 interaction with the negative regulatory receptor cytotoxic T lymphocyte‐associated protein 4 (CTLA‐4) inhibits the early activation of T cells.^14^, ^15^, ^16^ However, co‐stimulatory molecules like CD80, which are crucial for T cell activation, are often poorly expressed, whereas immunosuppressive molecules such as PDL1 and PDL2 are highly expressed on tumor and microenvironment cells.^17^ Therefore, low expression of CD80 may predict a poor prognosis in tumor patients.^18^

CD80 has been mainly used as an immunoadjuvant molecule in tumor or pathogenic microorganism vaccines to excite antigen‐specific T cells by transfection or co‐expression with antigens in vector cells.^19^ Tumor vaccines transfected with the CD80 gene restored tumor‐specific T cell activation and reversed PDL1‐mediated immunosuppression. Further, CD80 co‐expression inhibits the expression of PDL1 on the tumor cell surface.^20^

Recently, recombinant soluble CD80 (sCD80) containing the extracellular domains alone was used for tumor treatment because the binding affinity of CD80 to PDL1 was similar to its affinity with CD28.^3^, ^21^ The sCD80‐Fc fusion protein (sCD80 fused with an IgG Fc domain) showed greater therapeutic efficacy in preventing PD1:PDL1‐mediated suppression and restored T cell activation compared to treatment with monoclonal antibodies (mAb) against either PD1 or PDL1. sCD80‐Fc overcame PDL1‐mediated suppression in human or mouse tumor cells, facilitating T cell activation by binding to PDL1 to inhibit PDL1:PD1 interaction and by co‐stimulation via CD28.^22^, ^23^ Furthermore, sCD80‐Fc was able to prime T cells via the CD28 and non‐CD28 pathways in CD28‐knockout mice, in which CD80:CD28 binding was blocked.^24^ Therefore, sCD80 may serve as a promising antitumor factor as it primes active antitumor immunity by providing a co‐stimulatory signal and restores T cell activity by blocking the binding of PDL‐1 to PD‐1.^25^ It was reported that sCD80‐Fc delivered via intraperitoneal injection has a superior antitumor effect to PD‐1 antibodies in mice.^26^

Production of recombinant sCD80 protein using traditional in vitro expression methods has several disadvantages, such as high production costs and inconvenient administration. Therefore, it is necessary to explore new ways to produce checkpoint inhibitors.

The gastrointestinal tract is a suitable and economical bioreactor for biological drug generation and oral delivery. It is estimated that the total amount of biochemical metabolism in the gut equals the amount of liver metabolism.^27^ Lactic acid bacteria (LAB) and other probiotics are favorable carriers for the oral administration of immunotherapeutic drugs. In recent years, the use of live bacterial oral vaccine preparations or gene therapy using genetically engineered probiotics has been increasing. Probiotics are used to express pathogenic microorganism antigens, such as the rotavirus antigen and tetanus toxin C.^28^, ^29^ Bifidobacteria expressing cytosine deaminase have been used with 5‐fluorocytosine for tumor therapy via intravenous injection.^30^ In recent years, our group has used recombinant bifidobacteria to express several exogenous genes, such as oxyntomodulin, thymosin alpha, IL‐10, and tumstatin, for disease treatment in mice, resulting in positive curative effects.^31^, ^32^, ^33^, ^34^, ^35^ Importantly, the intestinal epithelium is involved in mucosal immunity besides serving as the main organ of nutrition and drug absorption, and may be useful for effective immunotherapy. Receptor‐mediated endocytosis is usually used to help recombinant proteins cross the intestinal mucosal barrier. Ligands, such as transferrin, M cell‐specific ligand, and cholera toxin B subunit (CTB), can bind to the receptors on the intestinal epithelium, thus aiding in the transportation of recombinant proteins across the barrier. CTB is a regulatory subunit of cholera toxin without intestinal toxicity, and the CTB receptor ganglioside M1 is highly expressed in the intestinal epithelium. Therefore, CTB is often used to guide the target protein across the intestinal mucosal barrier through receptor‐mediated endocytosis.^35^, ^36^, ^37^

In this study, we aimed to establish recombinant *Lactococcus lactis* carrying the human soluble CD80 (hsCD80) gene for colorectal cancer therapy. Additionally, recombinant *L. lactis* carrying the CTB‐hsCD80 fusion gene was also established to facilitate the free absorption of hsCD80 protein into blood circulation through the intestinal mucosal barrier by CTB‐mediated endocytosis for tumor therapy in other tissues.^38^, ^39^ The hsCD80‐transformed *L. lactis* showed therapeutic effects in primary colonic adenoma in APC^min/−^ mice, whereas the CTB‐hsCD80‐transformed *L. lactis* had pronounced effects on the growth of subcutaneous xenografts and melanoma lung metastases.

## 2 RESULTS

### 2.1 Recombinant protein expression in transformed *L. lactis* in vitro

**FIGURE 1:**
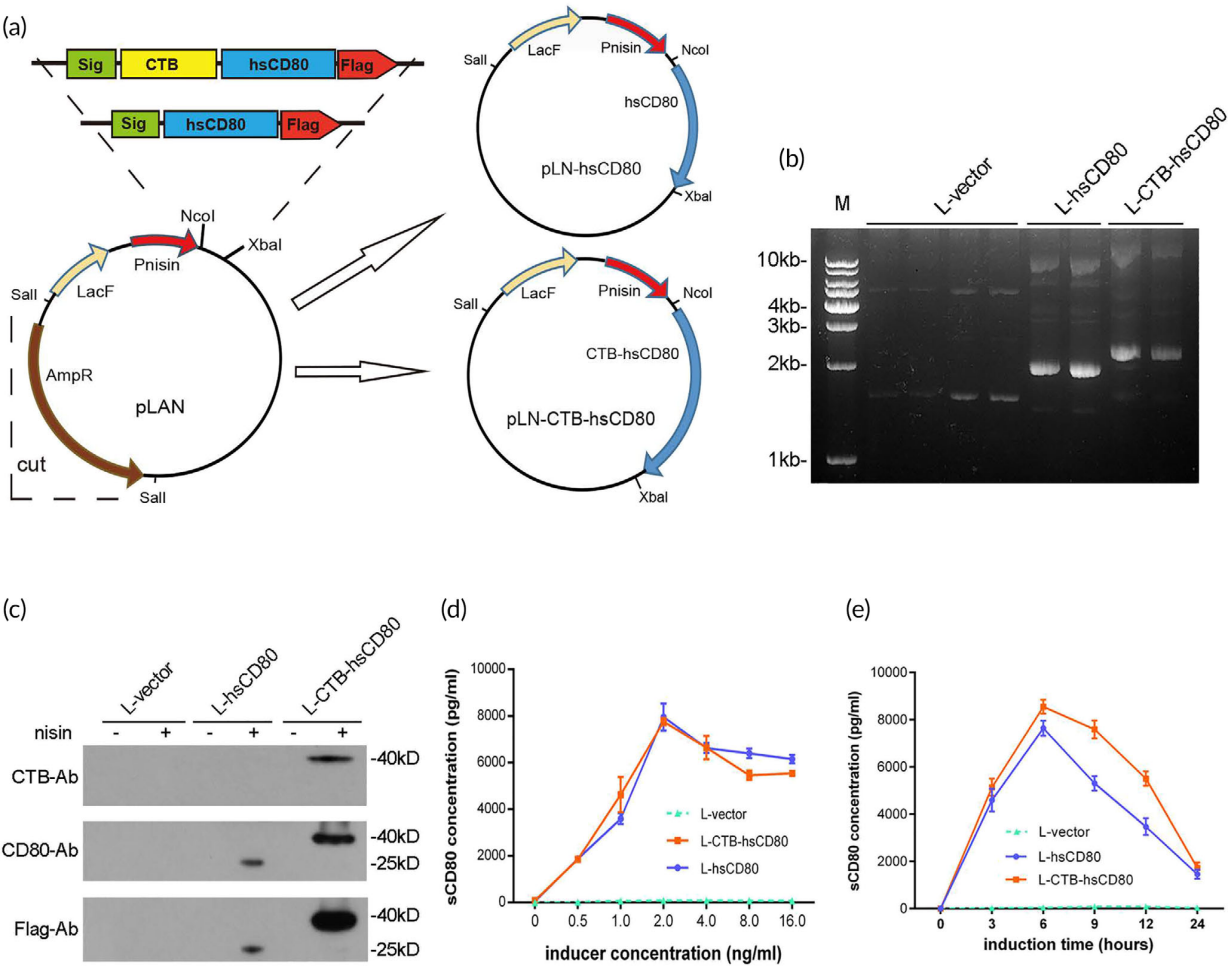
Identification and expression of *Lactococcus lactis* transformants in vitro. (a) Schematic of hsCD80 gene structure and plasmid construction. The hsCD80 gene or cholera toxin B (CTB)‐hsCD80 fusion gene with the SPK1 signal sequence was inserted into the *Escherichia coli‐L. lactis* shuttle vector pLAN at the *Nco*I and *Xba*I restriction sites. The food‐grade recombinant vectors pLN‐hsCD80 and pLN‐CTB‐hsCD80 were obtained by excision of the ampicillin resistance (AmpR) gene with the *Sal*I restriction enzyme. (b) Agarose gel electrophoresis analysis of plasmids extracted from L‐vector, L‐hsCD80, and L‐CTB‐hsCD80. (c) The expression of hsCD80 and CTB‐hsCD80 recombinant proteins was analyzed via western blotting. The proteins were detected with anti‐Flag, anti‐CD80, or anti‐CTB antibodies, respectively, after being induced by 2.0 ng/ml nisin for 6 h. (d) The expression of recombinant proteins with different inducer concentrations. (e) Expression of recombinant proteins at different induction times.

Plasmids extracted from selected colonies were identified by electrophoresis. The results showed that the plasmid sizes obtained were similar to the theoretical size of the recombinant plasmids pLN, pLN‐hsCD80, or pLN‐CTB‐hsCD80 (Figure 1a). The DNA sequencing results showed that the sequences detected were identical to those of the recombinant plasmids. The recombinant *L. lactis* strains transformed with pLN, pLN‐hsCD80, or pLN‐CTB‐hsCD80 were subsequently referred to as L‐vector, L‐hsCD80, and L‐CTB‐hsCD80, respectively. The protein expression and secretion from recombinant *L. lactis* cells were detected by western blot using anti‐Flag, anti‐CD80, or anti‐CTB antibodies (Abs) (Figure 1b). The results showed that the protein size of hsCD80 was ~25 kDa, whereas the size of CTB‐hsCD80 was ~40 kDa, which were similar to the molecular weights of the predicted proteins. The expression of recombinant CTB‐hsCD80 fusion protein was detected using three different Abs, whereas recombinant hsCD80 was detected using anti‐Flag and anti‐CD80 Abs after nisin induction. No recombinant protein expression was observed in the transformed *L. lactis* without nisin induction (Figure 1c). We analyzed the conditions for recombinant protein expression and secretion by recombinant *L. lactis* using different concentrations of the inducer (nisin) at different induction times. The results showed that the expression levels of recombinant hsCD80 or CTB‐hsCD80 protein reached a maximum when the concentration of nisin was 2 ng/ml. The expression level of recombinant protein reached a maximum at 6 h and subsequently showed a modest decrease when 2 ng/ml nisin was used to induce expression (Figure 1d,e).

### 2.2 Uptake of CTB‐hsCD80 fusion protein by intestinal epithelial cells in vitro

**FIGURE 2:**
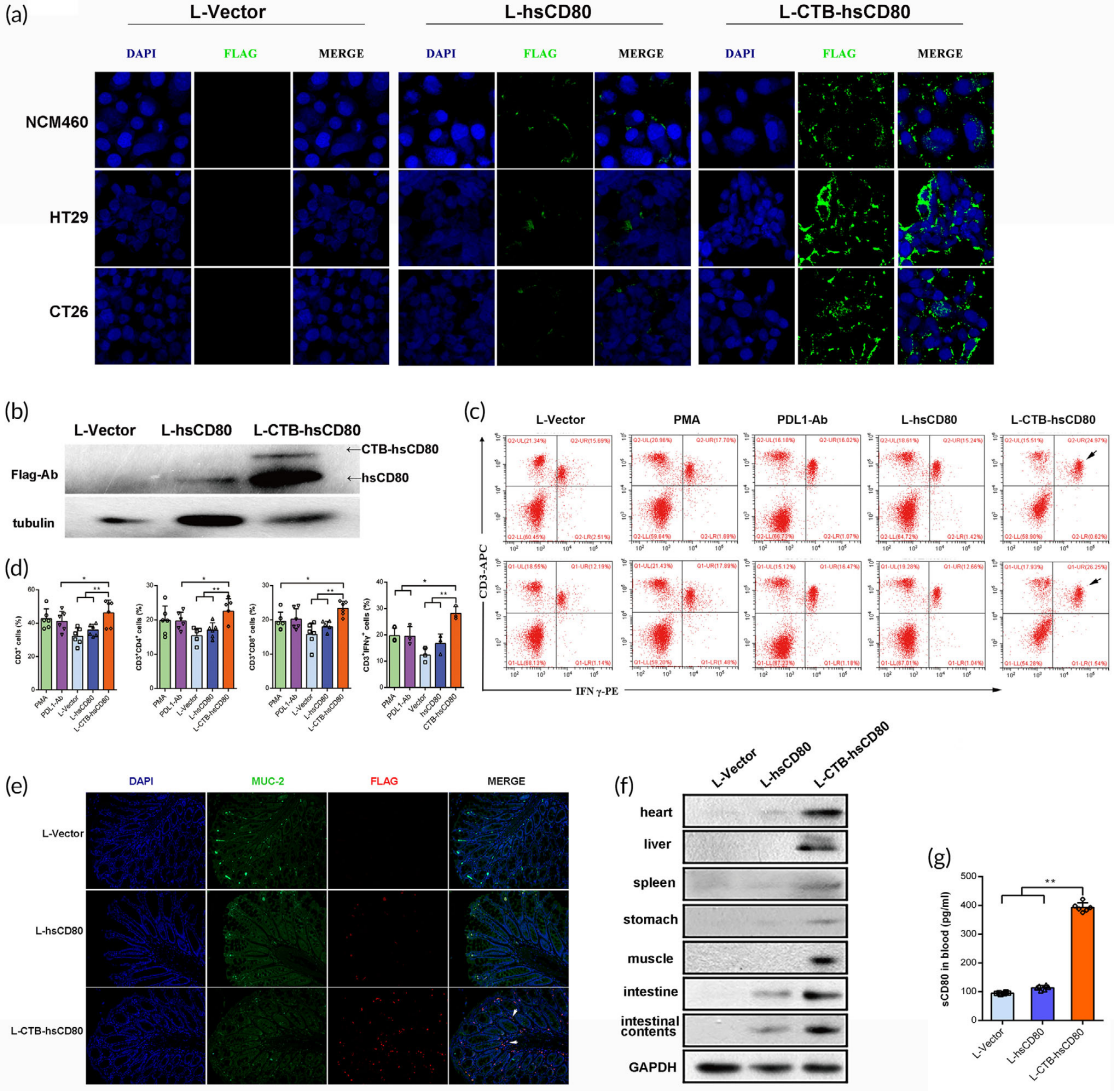
Uptake of the recombinant proteins expressed from *Lactococcus lactis* transformants was analyzed in intestinal epithelial cells in vitro. (a) The localization of recombinant proteins in normal human NCM460 intestinal epithelial cells, colon cancer HT29, and mouse colon cancer CT26 cells was evaluated via immunofluorescence analysis in vitro. Several cholera toxin B (CTB)‐hsCD80 proteins (green fluorescence) showed uptake into the cytoplasm. Green fluorescence indicates hsCD80 detected with anti‐Flag Ab. Blue fluorescence indicates the DAPI‐stained nucleus. (b) The recombinant CTB‐hsCD80 protein was digested and released as free hsCD80 after co‐incubation with colon cancer HT29 cells. (c) Scatterplot of CD3^+^IFN‐γ^+^ T cells analyzed using flow cytometry. The accumulation area of CD3^+^IFN‐γ^+^ cells is indicated with an arrow. (d) Percentage histogram of CD3^+^, CD3^+^CD4^+^, CD3^+^CD8^+^, and CD3^+^IFN‐γ^+^ cells. (e) Localization of the recombinant protein in the colon was visualized by immunofluorescence with anti‐Flag Ab. Recombinant protein (red fluorescence) was observed in the submucosal vascular area (white arrows). (f) The distribution of the recombinant hsCD80 protein in the intestinal contents, and the intestinal, muscle, cardiac, liver, and stomach tissues were detected via western blotting using anti‐Flag Ab. (g) The concentration of hsCD80 in the peripheral blood was detected using the hsCD80 ELISA kit. The data are representative of six independent experiments. Error bars indicate the mean ± SD. **p < 0.01.

We treated intestinal epithelial NCM460 cells, human colon cancer cell lines (CaCo2 and HT29), and mouse colon cancer CT26 cells with supernatant from L‐hsCD80 or L‐CTB‐hsCD80 transformants for 2 h. The recombinant proteins were evaluated for immunofluorescence (green) using the anti‐Flag Ab. The results showed that the CTB‐hsCD80 fusion protein from L‐CTB‐hsCD80 was endocytosed into different types of intestinal epithelial cells. However, low amounts of hsCD80 protein were endocytosed into the intestinal epithelial cells (Figure 2a).

The western blotting results showed that the CTB‐hsCD80 fusion protein was cleaved into the hsCD80 protein of ~25 kD after being incubated with intestinal epithelial cells. However, no small fragment of ~25 kD was observed when incubated with the acellular medium as the control (Figure 2b). The results indicated that the CTB‐hsCD80 fusion protein was digested specifically by furin protease within the cytoplasm and released free hsCD80 protein after uptake by intestinal epithelial cells, which depends on CTB receptor‐mediated endocytosis.

### 2.3 Proteins expressed from *L. lactis* transformants stimulate T cell activation and differentiation

After incubation with CaCo2 cells in the upper chambers of transwell plates for 2 h, the supernatant from recombinant *L. lactis* significantly affected the proportion of splenic lymphocyte subsets in the lower chambers. The results of the flow cytometry analysis showed that the proportion of CD3^+^CD4^+^, CD3^+^CD8α^+^, or CD3^+^ IFN‐γ^+^ T cell subsets in the L‐hsCD80 group was modestly greater than that of the L‐vector group. The proportion of CD3^+^CD4^+^, CD3^+^CD8α^+^, or CD3^+^ IFN‐γ^+^ T cell subsets in the L‐CTB‐hsCD80 group was significantly higher than that of the L‐vector or L‐hsCD80 groups, and higher than that of the PMA or PDL1‐mAb positive control groups (Figure 2c,d). The CTB‐hsCD80 fusion protein from recombinant *L. lactis* promoted the differentiation of T cells into CD3^+^CD4^+^ help T cells and promoted IFN‐γ secretion and the activation of T cells (IFN‐γ^+^CD3^+^) (Figure 2c,d).

### 2.4 CTB‐hsCD80 fusion protein crosses the intestinal mucosa and releases hsCD80 into the blood circulation in vivo

To verify whether the recombinant CTB‐hsCD80 protein expressed from the transformed *L. lactis* strains showed uptake by intestinal epithelial cells and enzymatically released free hsCD80 in the body, intestinal mucosal tissue sections were evaluated for immunofluorescence after the mice were administered the transformed bacteria by gavage for 1 week. The muc‐2 (green) fluorescence indicates the intestinal mucosa microvilli. The results of immunofluorescence analysis showed that CTB‐hsCD80 recombinant protein with a Flag tag (red) showed uptake by intestinal epithelial cells and reached the intestinal submucosal vascular area in the L‐CTB‐hsCD80 group. As a control, a small amount of recombinant protein (red) was observed on the surface of intestinal microvilli, and none in the submucous vascular area in the L‐hsCD80 treatment group. Moreover, no protein with a Flag tag (red) was observed on the intestinal microvilli surface and submucous vascular area in the L‐vector group (Figure 2e).

Free hsCD80 protein was detected in the intestine, stomach, liver, spleen, heart, and muscle tissues, as well as in the intestinal contents in the L‐CTB‐hsCD80 and L‐hsCD80 groups by western blotting. However, the hsCD80 protein was detected in the intestinal content, intestine, and heart of the L‐hsCD80 group alone. These results indicate that fusion expression with CTB enables hsCD80 to cross the intestinal mucosal barrier, enter the circulatory system, and reach various tissues and organs (Figure 2f,g).

### 2.5 Recombinant hsCD80 *L. lactis* completely suppresses primary intestinal adenoma growth and suppresses MDSCs in intestinal adenoma and intestinal mucosa

**FIGURE 3:**
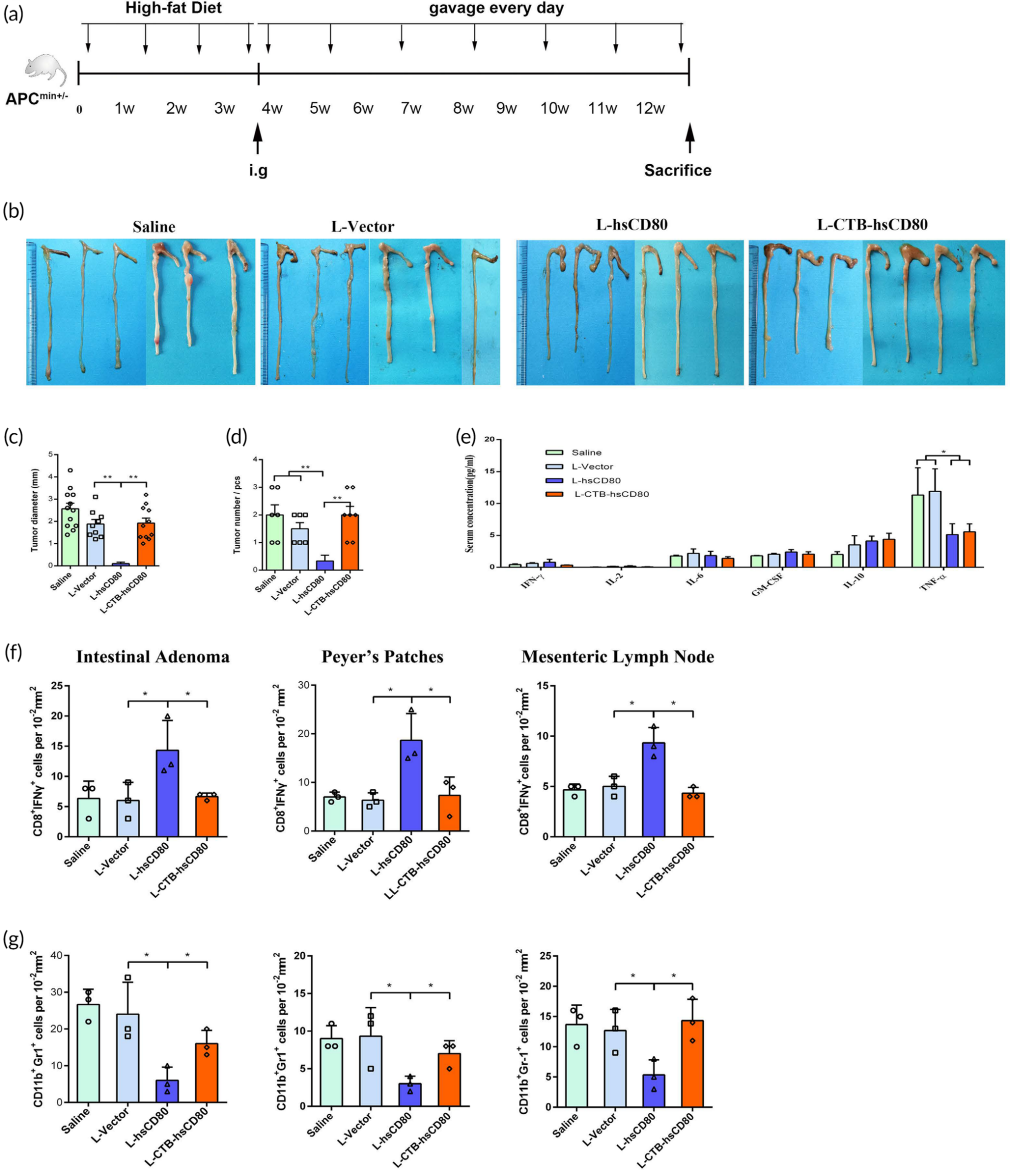
The inhibitory effect of engineered *Lactococcus lactis* on the growth of intestinal adenomas in antigen‐presenting cell (APC) mice. (a) Experimental design for the treatment of intestinal adenoma in APC mice in vivo. The mice were fed a high‐fat diet to accelerate the growth of intestinal adenomas for 3 weeks and were subsequently treated with 0.2 ml of 10^9^ CFU/ml engineered *L. lactis* containing the L‐vector, L‐hsCD80, or L‐cholera toxin B (CTB)‐hsCD80. (b) Macroscopic observation of colon adenoma after recombinant *L. lactis* treatment for 8 weeks. No adenoma was visible in the mouse colon in the L‐hsCD80 treatment group. (c, d) Statistical data showing the number and average diameter of adenomas in the colon. (e) The concentration of cytokines in the serum after recombinant *L. lactis* treatment. There was no statistical difference in the concentration of various cytokines in the serum after recombinant *L. lactis* treatment, except for a decrease in TNF‐α levels in the L‐hsCD80 and L‐CTB‐hsCD80 groups. (f) The effect of engineered *L. lactis* on CD3^+^IFN‐γ^+^ T cells in intestinal adenoma, Peyer's patches, and mesenteric lymph nodes in APC mice. (g) The effect of engineered *L. lactis* on CD11b^+^Gr‐1^+^ myeloid‐derived suppressor cells (MDSCs) in intestinal adenoma, Peyer's patches, and mesenteric lymph nodes in APC mice. Error bars indicate the mean ± SD. *p < 0.05.

The APC^min/−^ mice have a high susceptibility to developing intestinal adenomas spontaneously. In adult, APC^min/−^ heterozygous mice (at 8 weeks old) that are fed a high‐fat diet, multiple intestinal neoplasias (min) develop within 1 month. These mice are an ideal model for observing the impact of recombinant *L. lactis* on the growth of primary intestinal adenomas in the intestinal cavity. Male APC^min+/−^ mice were used to evaluate the efficacy of recombinant *L. lactis* against primary intestinal adenoma. The APC mice were sacrificed and necropsied after intragastric treatment with recombinant *L. lactis* for 8 weeks following 4 weeks of administering a high‐fat diet (Figure 3a). The results showed that the growth of primary intestinal adenoma was completely inhibited in the L‐hsCD80 group (*n* = 6). The adenomas were almost invisible in the intestinal tract with the naked eye (Figure 3b). Only two small adenomas of less than 0.5 mm in diameter were observed under the microscope. In the L‐CTB‐hsCD80 group (*n* = 7), the diameter of the intestinal adenoma was slightly smaller than that in the saline group. However, there was no significant difference in the number of adenomas between the L‐CTB‐hsCD80, saline, and the L‐vector groups (Figure 3c,d). The results indicate that the recombinant hsCD80 expressed from L‐hsCD80 may directly act on tumor cells and immune cells in the intestinal mucosa and restore cytotoxic T cell (TCL) activity by blocking PDL1/PD‐1 binding, resulting in a good antitumor effect. The recombinant L‐hsCD80 expressing *L. lactis* was more suitable for the treatment of primary intestinal tumors in comparison with the L‐CTB‐hsCD80 expressing *L. lactis*. There was no statistical difference in the concentration of cytokines in the serum after recombinant *L. lactis* treatment except for a decrease in TNF‐α levels in the L‐hsCD80 and L‐CTB‐hsCD80 groups (Figure 3e). CD8^+^IFNγ^+^ activated killer T cells and CD11b^+^Gr‐1^+^ myeloid‐derived suppressor cells (MDSCs) in intestinal adenoma, Peyer's patches, and mesenteric lymph nodes were evaluated via immunofluorescence analysis. The number of CD8^+^IFNγ^+^ cells in the intestinal adenoma, Peyer's patches, and mesenteric lymph nodes in the L‐hsCD80 group were increased significantly (Figure 3f, Supplementary Figures 1–3), which was higher than that in the L‐vector and L‐CTB‐hsCD80 groups, respectively (*p* < 0.05). The number of CD8^+^IFNγ^+^ cells in the L‐CTB‐hsCD80 group showed no significant differences compared with the L‐vector and saline groups. On the contrary, the number of CD11b^+^Gr‐1^+^ cells in the three tissues in the L‐hsCD80 group was significantly decreased (Figure 3g, Supplementary Figures 4–6), which was lower than that in the L‐vector and L‐CTB‐hsCD80 groups, respectively. The numbers of CD11b^+^Gr‐1^+^ cells in intestinal adenoma and Peyer's patches in the L‐CTB‐hsCD80 group were slightly lower than that in the L‐vector and saline groups. However, there was no significant difference between the three groups.

### 2.6 Recombinant *L. lactis* suppresses the growth of melanoma lung metastases

**FIGURE 4:**
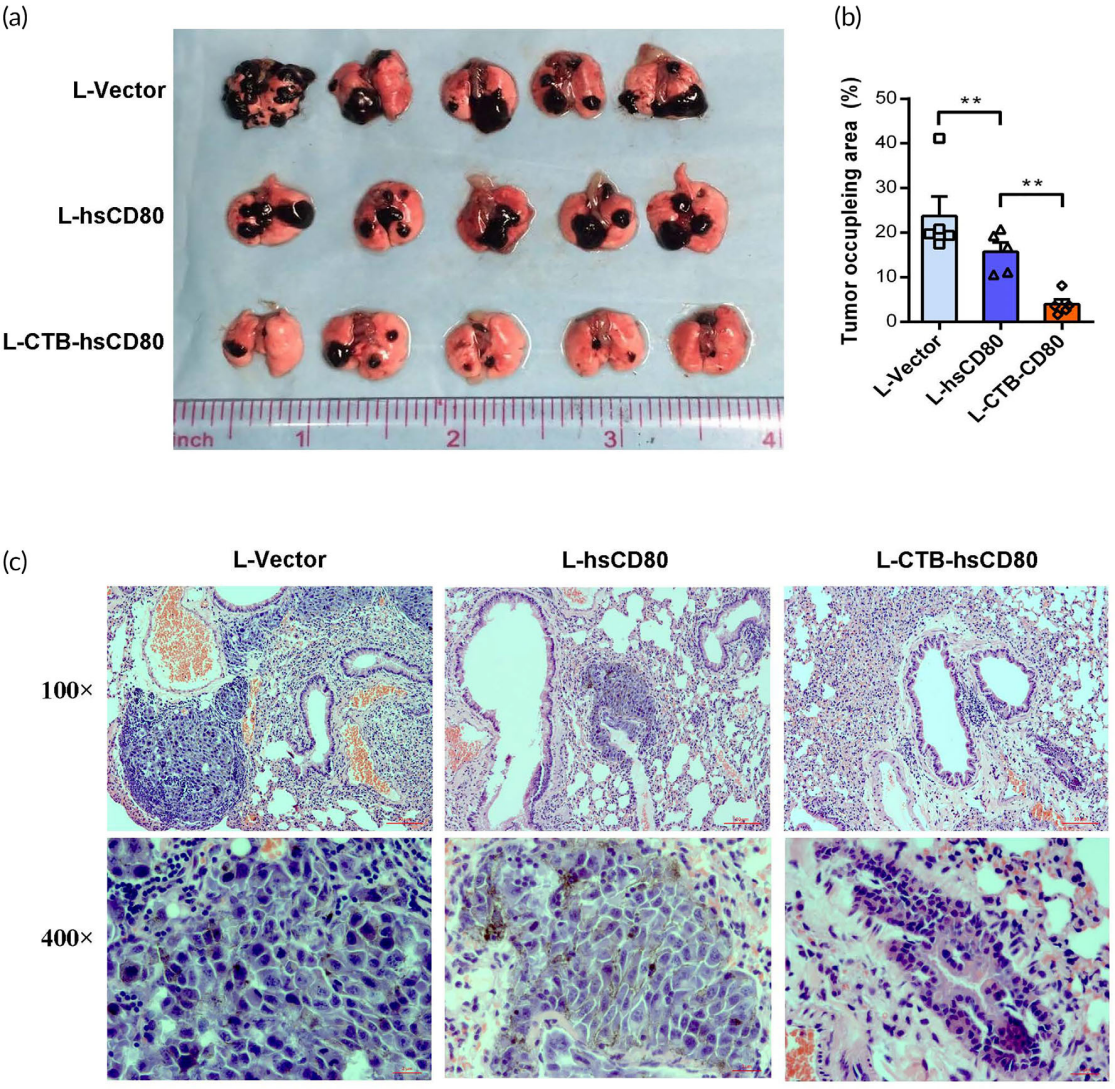
The inhibitory effect of engineered *Lactococcus lactis* on the growth of melanoma lung metastases in BALB/c mice. (a) Macroscopic observation of melanoma lung metastases after recombinant *L. lactis* treatment. (b) Statistical data for the area occupied by melanoma metastasis nodules in the lung. (c) Hematoxylin–eosin staining of lung tissue with melanoma metastatic tumors.

The melanoma lung metastasis model was used to evaluate the inhibitory effect of recombinant *L. lactis* on tumor metastasis. After treatment with recombinant *L. lactis* for 21 days, all tumor‐bearing mice were euthanized and necropsied. The number of visible lung metastatic nodules and the volumetric fraction occupied by metastatic melanoma were quantified. The lung metastatic tumor images are shown in Figure 4a. The number and size of melanoma metastatic tumors on the lung surface in the L‐hsCD80 group were significantly lower than those in the L‐vector group. The number and size of melanoma metastases in the L‐CTB‐hsCD80 group were significantly lower than those of the L‐hsCD80 and L‐vector groups. Although 23.70 ± 9.83% of the lungs were occupied by melanoma in the L‐vector control group, only 15.68 ± 4.65% and 3.97 ± 2.47% of the lung area was occupied in the L‐hsCD80 and L‐CTB‐hsCD80 groups, respectively. There were significant differences between the L‐CTB‐hsCD80 and L‐hsCD80 groups (*p* = 0.001), and between the L‐CTB‐hsCD80 and L‐vector control groups (*p* = 0.0001) (Figure 4b). The results suggest that L‐CTB‐hsCD80 has a greater inhibitory effect on the growth of melanoma metastases than L‐hsCD80. Pathological analysis can often mistake metastatic tumors for tissue inflammation; thus, in Figure 4c, we show both a 100× and 400× enlarged image to avoid confusion. The 100× image shows the approximate distribution of B16F10 melanoma metastasis in the lung tissue of mice. The 400× image displays more detailed pathological features of melanoma metastasis and is meant to confirm that the abnormal structure in the lung tissue is B16F10 metastasis rather than lymphocyte infiltration. These images suggest that L‐CTB‐hsCD80 has a greater inhibitory effect on the growth of melanoma metastases than L‐hsCD80 at the pathological level.

### 2.7 hsCD80 recombinant *L. lactis* evokes active immunity against colon cancer

**FIGURE 5:**
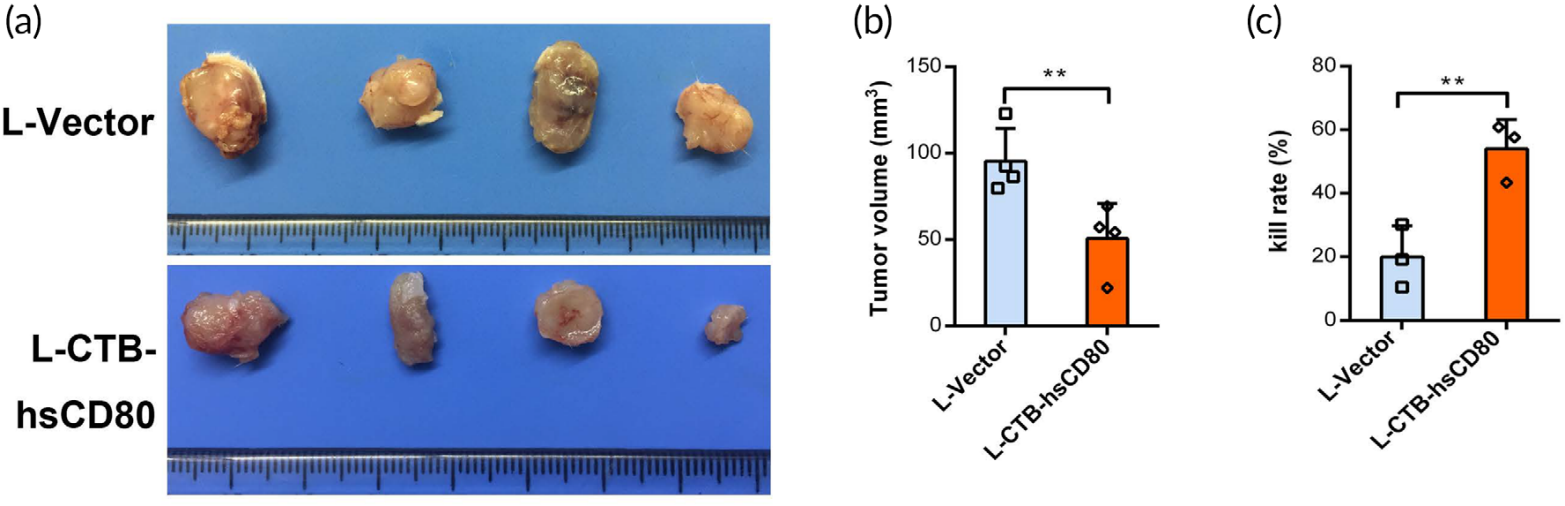
The active immune effect of cholera toxin B (CTB)‐hsCD80 fusion protein derived from engineered *Lactococcus lactis* on the growth of subcutaneous xenografts with the mouse colon cancer CT‐26 strain. (a) The xenografts of colon cancer CT26 cells co‐incubated with the expression supernatant of recombinant *L. lactis* for 2 h before subcutaneous injection. (b) Graph showing the tumor volume of colon cancer xenografts. (c) The kill rate of active T cells activated with the expression supernatant of recombinant *L. lactis* in vitro. The data are representative of each group of mice or independent experiments. Error bars indicate the mean ± SD. *p < 0.05; **p < 0.01.

To ascertain whether the CTB‐hsCD80 fusion protein can stimulate active immunity after endocytosis by colon cancer cells, the specific killing effect of lymphocytes activated by the CTB‐hsCD80 protein expressed from recombinant *L. lactis* on colon cancer cells in vivo and in vitro was analyzed. After co‐incubation with supernatant from L‐CTB‐hsCD80 transformants for 2 h before CT26 cell transplantation into mice, the volume of the transplanted tumor was found to be significantly smaller than that of the L‐vector group (*n* = 4) (Figure 5a,b).

Co‐incubation of CT26 cells with lymphocytes pretreated with the supernatant from recombinant *L. lactis* showed that the killing rate of lymphocytes to tumor cells in the L‐CTB‐hsCD80 group was significantly higher than that of the L‐vector group (Figure 5c). Thus, colon cancer cells ingesting the CTB‐hsCD80 fusion protein from the supernatant of recombinant *L. lactis* may act as a tumor vaccine and stimulate active antitumor immunity by releasing free hsCD80 protein.

### 2.8 Recombinant *L. lactis* suppresses the growth of subcutaneous xenografts

**FIGURE 6:**
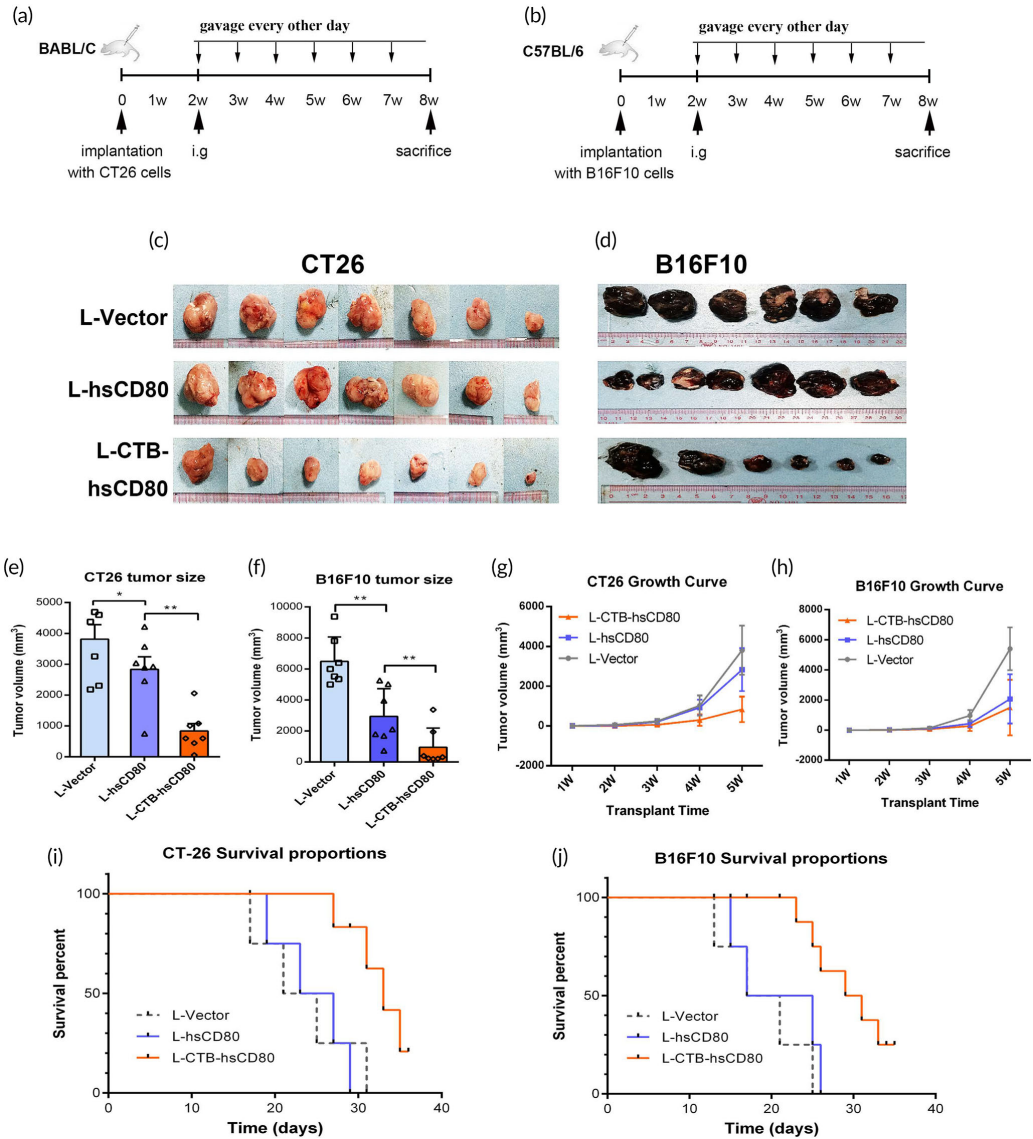
The inhibitory effect of engineered *Lactococcus lactis* on the growth of subcutaneous colon cancer and melanoma xenografts. (a, b) Experimental design of the treatment of subcutaneous colon cancer and melanoma xenografts in vivo. The arrows indicate the injection of 1 × 10^6^ mouse colon cancer CT26 cells or B16F10 melanoma cells, and the intragastric administration of recombinant *L. lactis*. (c, d) The tumor size of colon cancer or melanoma xenografts after recombinant *L. lactis* treatment for 6 weeks. (e, f) Statistical data for the tumor volumes of colon cancer or melanoma xenografts. (g, h) Growth curves of mice bearing colon cancer or melanoma xenografts, respectively. (i, j) Survival curves of mice bearing colon cancer or melanoma xenografts, respectively. Data are representative of each group of mice (The survival date was calculated from the day of starting i.g with recombinant bacteria). Error bars indicate the mean ± SD. *p < 0.05; **p < 0.01.

To assess the therapeutic effect of the oral delivery of recombinant *L. lactis* on tumors in other tissues outside the intestinal epithelium, tumor‐bearing mice with subcutaneous xenografts of colon cancer CT26 or melanoma cancer B16F10 cells were orally administered recombinant bacteria (Figure 6a,b). For mice with either CT26 or B16F10 xenografts, the average tumor volume in the L‐CTB‐hsCD80 group was significantly smaller than that in the L‐hsCD80 and L‐vector groups. The average tumor volume in the L‐hsCD80 group was slightly smaller than those in the L‐vector group (Figure 6c–f, Supplementary Figure 7). However, there was no statistical difference between the two groups. Growth curves of mice bearing colon cancer or melanoma xenografts were shown in Figure 6g,h, respectively. The survival rate of tumor‐bearing mice in the L‐CTB‐hsCD80 group was significantly higher than that of the L‐hsCD80 and L‐vector groups in both xenograft models administered CT26 and B16F10 cells (Figure 6i,j).

### 2.9 Recombinant *L. lactis* inhibits tumor cell proliferation and angiogenesis

**FIGURE 7:**
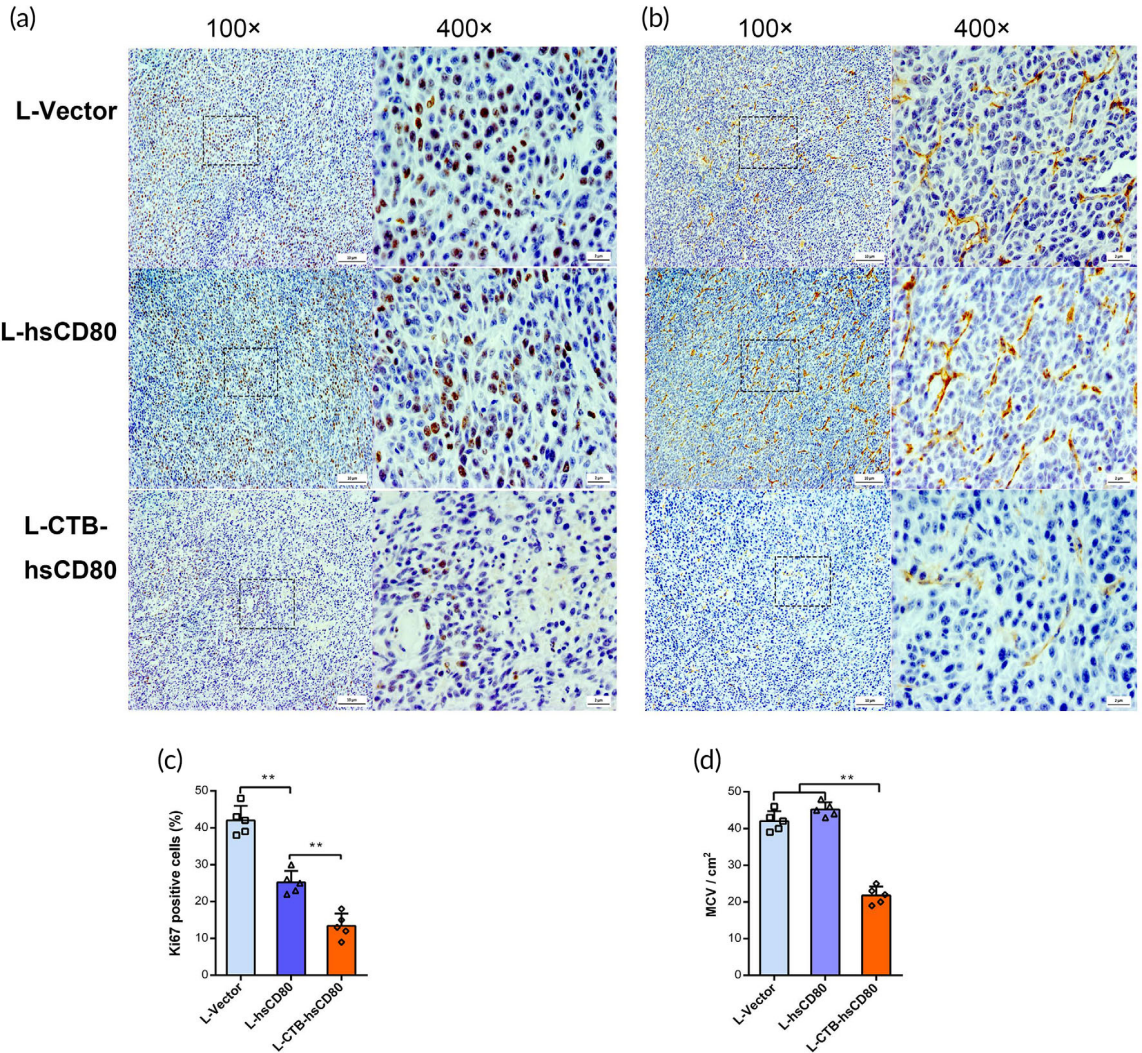
Pathological analysis of tumor proliferation and angiogenesis by immunohistochemical staining. (a) Ki‐67‐positive proliferative cells in CT26 transplanted tumor sections. (b) Angiogenesis was evaluated using CD31 positive staining in CT26 cell‐transplanted tumors. (c) The percentage of Ki‐67‐positive cells in transplanted tumors. (d) Statistical analysis of microvessel density (MVD) in transplanted tumors shows the area occupied by CD31^+^ cells relative to the total area of the tumor cells. Data are representative of images obtained from five independent sections. Error bars indicate the mean ± SD. *p < 0.05; **p < 0.01.

To understand the effect of recombinant *L. lactis* on tumor cell proliferation and angiogenesis, the expression levels of Ki‐67 and CD31 in tumor tissues were analyzed via immunohistochemistry. The hematoxylin–eosin staining of the CT26 xenograft sections showed that the tumor cell density was decreased, tissue fibrosis was observed, and the ratio of cell nucleus/cytoplasm decreased significantly in the L‐CTB‐hsCD80 group. However, the cell density was slightly decreased, and a modest amount of tissue fibrosis appeared in the L‐hsCD80 group in comparison with the L‐vector group (Supplementary Figure 8). The number of Ki‐67 positive cells in the tumor decreased significantly in the L‐CTB‐hsCD80 group, whereas the number of Ki‐67 positive cells was modestly decreased in the L‐hsCD80 group compared with the L‐vector group (Figure 7a,c).

In the L‐CTB‐hsCD80 group, the microvessel density (MVD) using CD31‐positive staining was significantly decreased, and there was no difference in MVD between the L‐hsCD80 and L‐vector groups (Figure 7b,d).

The TUNEL (green) assay immunofluorescence indicated the number of apoptotic cells in the tumor. The results showed that the number of apoptotic cells in the tumor sections of the L‐CTB‐hsCD80 group was significantly higher than that of the L‐hsCD80 and L‐vector groups. The proportion of apoptotic cells was 3.93% in the L‐CTB‐hsCD80 group and 1.31 and 1.06% in the L‐hsCD80 and L‐vector groups, respectively (Supplementary Figure 9). Taken together, the results indicate that oral administration of recombinant *L. lactis* L‐CTB‐hsCD80 may induce tumor cell apoptosis and inhibit cell proliferation and angiogenesis of the xenografts.

### 2.10 Recombinant *L. lactis* promotes T cell activation and differentiation in the spleen and tumor

**FIGURE 8:**
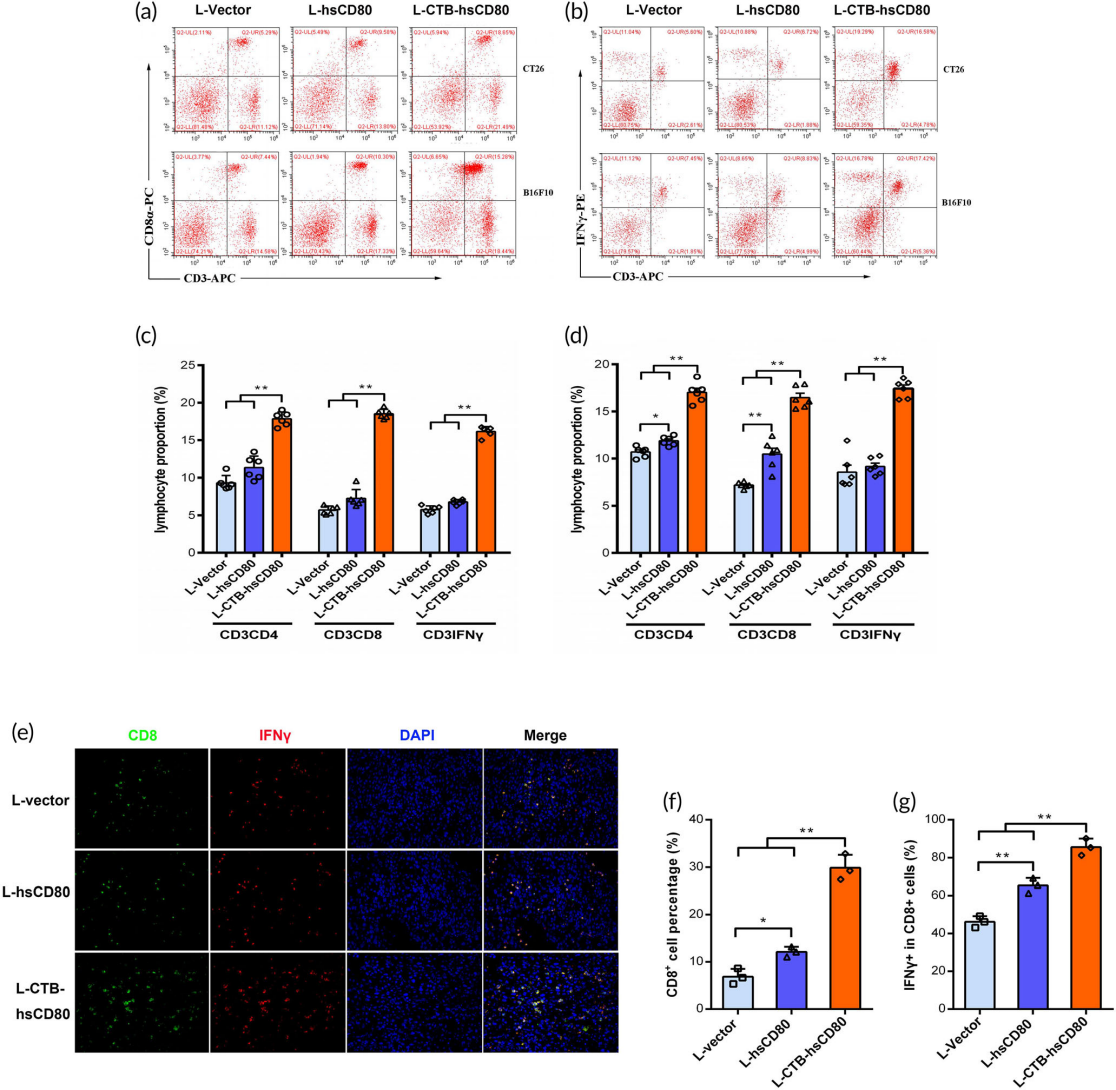
The proportion of splenic mononuclear cells after oral treatment with recombinant *Lactococcus lactis* in vivo. (a) Scatterplot of CD3^+^CD8^+^ lymphocytes in mice bearing subcutaneous transplantation tumors that were identified via flow cytometry. (b) Scatterplot of CD3^+^IFNγ^+^ in splenic mononuclear cells. (c) The percentage of CD3^+^CD4^+^, CD3^+^CD8^+^, and CD3^+^IFNγ^+^ cells in splenic mononuclear cells in mice bearing CT26 transplantation tumors. (d) The percentage of CD3^+^CD4^+^, CD3^+^CD8^+^, and CD3^+^IFNγ^+^ cells in splenic lymphocytes in mice bearing melanoma B16F10 xenografts. (e) CD8^+^IFNγ^+^ T cells in CT26‐transplanted tumor sections. (B) The percentage of CD3^+^CD8^+^ T cells in the transplanted tumor sections. (f) The percentage of IFN‐γ^+^ cells among CD8^+^ T cells in transplanted tumor sections. The data are representative of three independent images. Error bars indicate the mean ± SD. *p < 0.05; **p < 0.01.

The results of flow cytometry analysis in CT26 xenograft‐bearing mice showed that the proportion of CD3^+^CD4^+^, CD3^+^CD8α^+^, or IFN‐γ^+^CD3^+^ T cell subsets in the spleen in the L‐hsCD80 group was higher than that of the L‐vector group. Furthermore, the proportion of CD3^+^CD4^+^, CD3^+^CD8α^+^, or IFN‐γ^+^ CD3^+^ T cell subsets in the L‐CTB‐hsCD80 group was significantly higher than that of the L‐vector or L‐hsCD80 group. Similar results were obtained in B16F10 xenograft‐bearing mice (Figure 8c,d).

The CD8^+^ and IFN‐γ^+^ infiltrating lymphocytes within the local tumor microenvironment were identified via immunofluorescence analysis using anti‐CD8 (green) and anti‐IFN‐γ Ab (red) to ascertain whether the hsCD80‐transformed *L. lactis* promotes T lymphocyte activation and antitumor immunity. In Figure 8e, the number of CD8 and IFN‐gamma double‐positive cells in the L‐CTB‐hsCD80 group increased, and the expression levels of CD8 and IFN‐gamma in most lymphocytes increased slightly (exhibiting higher fluorescence intensity). However, the variance between different activated lymphocytes was large after L‐hsCD80 and L‐CTB‐hsCD80 treatment. (Figure 8e), and most CD8^+^ cells coincided with IFN‐γ^+^ cells. Over 65% of CD8^+^ cells had high IFN‐γ + expression in the LL‐sCD80 group, which was significantly higher than that of the L‐vector group (46.14%). Furthermore, the percentage of CD8^+^ T cells with high IFN‐γ^+^ expression in the LL‐CTB‐sCD80 group (85.56%) was significantly higher than that in the LL‐sCD80 and L‐vector groups. Additionally, the percentage of IFN‐γ^+^ CD8^+^ cells relative to the total cells in the LL‐CTB‐sCD80 group (29.84%) was significantly higher than that in the sCD80 (12.12%) and L‐vector (6.87%) groups (Figure 8f,g).

The results indicate that hsCD80‐transformed *L. lactis* promotes the differentiation and maturation of splenic lymphocytes and activates infiltrating lymphocytes within the transplanted tumor. CTB‐sCD80 has a better effect on activating lymphocytes, as it crosses the intestinal mucosal barrier and enters the circulation more easily.

## 3 DISCUSSION

Recently, immune checkpoint inhibitors, such as anti‐PD‐1 and anti‐PDL1 Abs, have been widely used in cancer therapy. However, anti‐PD‐1 or anti‐PDL1 Ab as a single agent is not sufficient to improve clinical outcomes in most patients.^39^, ^40^ Combination therapies with anti‐PD‐1 and anti‐CTLA‐4 Abs increased the response rate but resulted in severe immune‐associated adverse events.^41^, ^42^ Combination therapy with PD‐1 blocking and OX40 agonist Abs induced T cell apoptosis in the periphery and the tumor, resulting in adverse antitumor effects.^43^ Furthermore, PD‐1 blockade treatment before tumor vaccine priming can induce subprimed CD8^+^ cells converted into dysfunctional PD‐1^+^CD38^hi^ cells and interfere with the therapeutic outcomes. Sequencing anti‐PD‐1 and vaccines is crucial for successful therapy. The number of dysfunctional PD‐1^+^CD38hi cells was reduced, resistance to PD‐1 Ab was reversed, and better curative effects were obtained when the tumor vaccine was administered before the PD‐1 blocking Ab.^44^ Reportedly, soluble CD80 (sCD80) evokes antitumor immunity through CD28 and non‐CD28 pathways and restores T cell activity by blocking PD‐1: PDL1 binding, exhibiting an improved antitumor effect than PD‐1 and PD‐L1 Abs.^23^, ^24^

Considering the high cost and inconvenience of recombinant CD80 injection, we constructed recombinant *L. lactis* bearing the hsCD80 gene for colorectal cancer therapy. *L. lactis* is a safer host bacterium for foreign genes compared with *Escherichia coli* for several reasons. First, it does not produce endotoxin or exotoxin, making it safer. Second, the recombinant protein expressed by *L. lactis* can be secreted outside the bacteria, whereas *E. coli* can only secrete the recombinant exogenous polypeptide into the periplasm under the guidance of secretory signal peptides, because it is Gram‐negative. Third, *L. lactis* can grow and colonize in the intestine, continuously expressing and secreting recombinant protein into the intestinal cavity. This not only improves the local efficacy of recombinant protein drugs, but also greatly reduces the production cost and frequency of administration. Therefore, *L. lactis* is particularly suitable for the treatment of malignant intestinal tumors and other gastrointestinal diseases. Although recombinant bifidobacteria and LAB can be injected directly into the tumor nodes to attack the tumor, this approach has limitations in clinical practice. It is not suitable for small tumors, advanced tumors with multiple invasive and metastatic lesions, or treatment after tumor resection.

Additionally, the CTB‐hsCD80 fusion gene containing *L. lactis* was established to facilitate CTB‐hsCD80 absorption by the intestinal epithelium and the release of free hsCD80 protein into the blood circulation for tumor therapy in other organs. CTB is a regulatory subunit of cholera toxin without intestinal toxicity. The CTB receptor ganglioside M1 shows high expression in the intestinal epithelium. CTB is often used to guide the target protein to cross the intestinal mucosal barrier through receptor‐mediated endocytosis. Fusion expression of green fluorescent protein (GFP), IFN‐α, or myelin basic protein with CTB in tobacco significantly improved the absorption of GFP and IFN‐α into the blood circulation after oral administration.^35^, ^38^ Our results showed that the hsCD80 and CTB‐hsCD80 fusion proteins were expressed and secreted from recombinant *L. lactis* after nisin induction in vitro or in vivo. The CTB‐hsCD80 fusion protein from L‐CTB‐hsCD80 was endocytosed into the cytoplasm, and free hsCD80 was released after co‐incubation with different types of intestinal epithelial cells. In vivo analyses showed that the Flag‐labeled recombinant CTB‐hsCD80 protein could enter the submucosal vascular area of the intestine and was detectable in the liver, heart, muscle, and other tissues after oral administration of live recombinant bacteria. These results demonstrate that the CTB‐hsCD80 fusion protein secreted from recombinant *L. lactis* showed uptake by intestinal epithelial cells through CTB receptor‐mediated endocytosis, and released free hsCD80 protein into the blood circulation.

The supernatant of recombinant *L. lactis* co‐incubated with CaCo2 cells in the upper chambers of transwell plates significantly increased the proportion of CD3^+^CD4^+^, CD3^+^CD8α^+^, or CD3^+^ IFN‐γ^+^ primed T cell subsets. Moreover, the co‐incubation of the CTB‐hsCD80 expression supernatant significantly enhanced the killing effect of lymphocytes on tumor cells in vitro. The growth of the CT26 xenograft was significantly inhibited by pretreatment with the CTB‐hsCD80 expression supernatant. These results suggest that the endocytosis of the CTB‐hsCD80 protein into colon cancer cells can serve as a live tumor vaccine to stimulate antitumor active immunity.

The results of immunotherapy in APC mice showed that recombinant L‐hsCD80 *L. lactis* had significant inhibitory effects on the growth of primary colorectal adenoma after oral administration. The growth of colorectal adenomas was completely inhibited by recombinant L‐hsCD80 treatment. The results of immunofluorescence analysis on local immune cells showed that CD8^+^IFNγ^+^ activated cytotoxic T cells (TCLs) were increased in intestinal adenomas, Peyer's patches, and mesenteric lymph nodes whereas CD11b^+^Gr‐1^+^ MDSCs were decreased in the L‐hsCD80 group. Our results indicate that recombinant L‐hsCD80 *L. lactis* has a good therapeutic effect on the primary intestinal mucosa by directly acting on the tumor and promoting mucosal immunity. We reasoned that the recombinant hsCD80 expressed from L‐hsCD80 may directly block PD‐1:PDL1 binding on local intestinal adenomas. Moreover, it directly activates TCL and inhibits MDSCs in the intestinal mucosa to enhance mucosal immunity, resulting in a prominent therapeutic effect. The efficacy of recombinant L‐CTB‐hsCD80 is less than that of L‐hsCD80, and this may result because the CTB‐hsCD80 fusion protein interferes with the structure of hsCD80 and reduces its immune activity.

Analysis of orally administered live recombinant *L. lactis* in CT26 and B16F10 xenograft‐bearing mice showed that the proportion of primed T cells and activated CD8^+^IFNγ^+^ TCLs was significantly increased. These results are consistent with previously reported results using recombinant sCD80‐Fc in vitro or in vivo.^21^, ^22^

Further, recombinant L‐CTB‐hsCD80 *L. lactis* was more efficient than L‐hsCD80 recombinant bacteria in inhibiting the growth of colon cancer and melanoma xenografts, and significantly prolonged the life of tumor‐bearing mice. The results of the proliferation marker analysis using Ki‐67, CD31‐labeled MVD, and apoptosis via TUNEL assays showed that recombinant L‐CTB‐hsCD80 had better inhibitory effects on xenograft growth than L‐hsCD80. We inferred that the efficacy difference between the two recombinant *L. lactis* may result because the CTB‐hsCD80 protein expressed from recombinant L‐CTB‐hsCD80 easily passes through the intestinal mucosal barrier and releases free hsCD80 into the blood circulation. However, hsCD80 expressed from L‐hsCD80 rarely crosses the intestinal mucosal barrier, and instead acts on the local immune cells of the intestinal mucosa and plays an antitumor role. The number of activated CD8^+^IFNγ^+^ T cells in tumor‐infiltrating lymphocytes in the xenograft after L‐CTB‐hsCD80 treatment increased significantly, whereas the number of activated T cells modestly increased after L‐hsCD80 treatment, supporting our inference.

We showed that the administration of recombinant *L. lactis* by oral delivery does not cause serious harm to experimental mice. There was no statistical difference in the liver and kidney organ indexes between the different groups. Although TNF‐α concentrations in the serum decreased in the L‐CTB‐hsCD80 and L‐hsCD80 groups, the concentrations of IL‐2, IL‐6, and other cytokines did not change significantly. These data indicate that oral administration of recombinant *L. lactis* does not trigger an abnormal immunological response and showed no toxicity to tumor‐bearing mice. Furthermore, the recombinant proteins are continuously and stably expressed from recombinant *L. lactis* in the intestinal cavity, and only a small amount of free hsCD80 enters the blood circulation and maintains a relatively low effective concentration, which ensures the safety of the medication. In short, this is a safe drug or method for tumor therapy.

**FIGURE 9:**
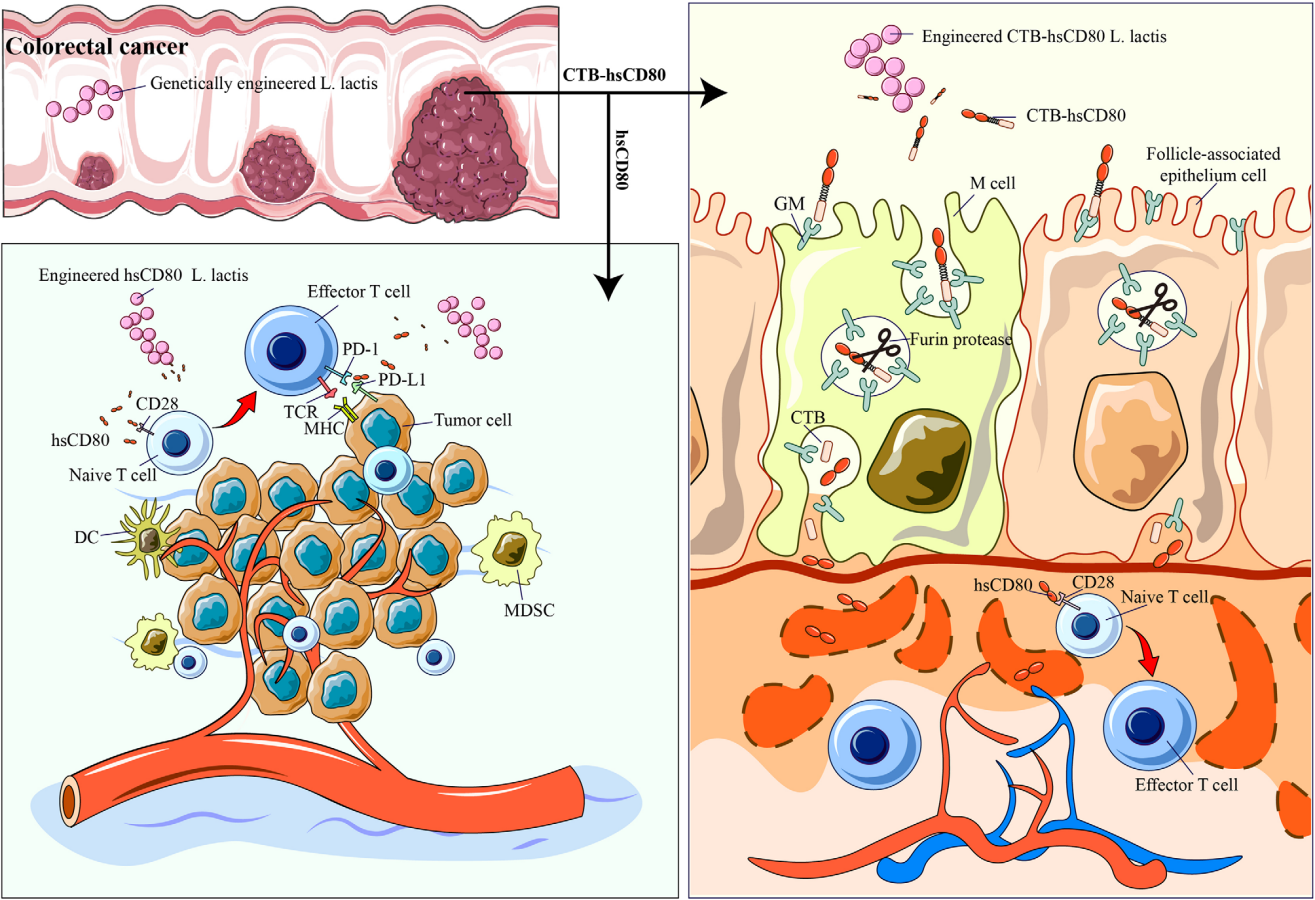
Enhancing antitumor immunity and suppressing tumor growth through oral delivery of soluble CD80 by recombinant Lactococcus.

To conclude, we established a food‐grade LAB expression system for sCD80 expression and tumor immunotherapy. Through fusion expression with CTB, sCD80 can cross the intestinal mucosal barrier and enter the blood circulation to play a systemic antitumor effect. Recombinant sCD80 from *L. lactis* can exert antitumor effects by blocking PD‐1/PDL‐1 binding, triggers and activates TCLs, and is activated by tumors to form a live vaccine to stimulate active immunity (Figure 9). The effect on primary colorectal cancer is particularly notable. Recombinant LAB are also suitable for multipathway and multidrug combined antitumor therapy, which is important for most refractory malignancies. Thus, the method outlined in this study may provide a new antitumor immunotherapeutic approach for the treatment of malignant tumors, which is simple to manufacture, easy to use, and safe.

## 4 MATERIALS AND METHODS

### 4.1 Plasmids, bacterial strains, cell lines, and mice

The nisin‐controlled gene expression system vector pNZ8148 and *L. lactis* NZ3900 were obtained from MoBiTec GmbH (Goettingen, Germany). *L. lactis* was cultured anaerobically in Elliker medium at 30°C according to the manual. The cell lines for normal human colonic epithelial cells NCM460, human colon cancer cells CaCo2, HT29, SW480, and SW1116, mouse colon cancer cells CT‐26, and mouse melanoma cells B16‐F10 were obtained from the Department of Cell Biology, Southern Medical University. The cells were cultured in Dulbecco's modified Eagle's medium (DMEM, GIBCO), supplemented with 10% fetal bovine serum (GIBCO), 50 U/ml penicillin, and 50 mg/ml streptomycin in a 5% CO_2_ humidified atmosphere at 37°C.

All animal experiments were approved by the Ethics Committee for Animal Care of Southern Medical University (China), and the animal facility was approved by the National Association of Laboratory Animal Care. Pathogen‐free 8‐week‐old female BALB/c mice were obtained from the Experimental Animal Center of Southern Medical University, China. Pathogen‐free 8‐week‐old male APC^min/+^ (C57BL/6J‐ApcMin/NJU) mice were obtained from the Model Animal Center of Nanjing University, China. All mice were bred under SPF conditions, and they had free access to food and water.

### 4.2 Vector construction

The scheme of the food‐grade expression vector construction is shown in Figure 1. The hsCD80 and CTB‐hsCD80 fusion genes were designed and synthesized with the SPK1 signal peptide fused at the N‐terminal. A furin protease cleavage sequence (Arg‐Ala‐Arg‐Arg) was added between CTB and hsCD80 to facilitate enzymatic hydrolysis within intestinal mucosal cells and the release of free hsCD80. To improve the target gene expression efficiency, DNA sequences were optimized based on the preference codon of *L. lactis*. To facilitate detection and distinguish heterologous expression of hsCD80 from endogenous hsCD80, a Flag tag was introduced C‐terminal to hsCD80. The hsCD80 gene and the CTB‐hsCD80 fusion gene were inserted into the pLAN expression plasmid, an *E. coli*‐*L. lactis* shuttle vector derived from pNZ8148 with a resectable β‐lactamase gene, at the *Nco*I and *Xba*I restriction sites. Food‐grade recombinant vectors pLN‐hsCD80, pLN‐CTB‐hsCD80, and the control empty vector pLN were obtained by excising the ampicillin resistance gene with the *Sal*I restriction endonuclease (Figure 1a). All recombinant plasmids were verified by DNA sequencing (Shangon Biotech, China).

### 4.3 Gene transformation and recombinant bacterial identification

The recombinant plasmids pLN‐hsCD80, pLN‐CTB‐hsCD80, or empty vector pLN were transformed into *L. lactis* NZ3900 by electroporation using a Gene Pulser and Pulse Controller apparatus (Bio‐Rad, USA) at 2.5 kV, 25 μF, and 200 Ω as described previously.^30^ The transformed *L. lactis* were selected using Elliker medium with lactose as the sole carbon source. Positive colonies were identified by PCR, restriction enzyme digestion, and DNA sequencing. The recombinant *L. lactis* transformed with pLN, pLN‐hsCD80, or pLN‐CTB‐hsCD80 was subsequently referred to as L‐vector, L‐hsCD80, and L‐CTB‐hsCD80, respectively.

### 4.4 Recombinant protein expression analysis in vitro

The transformed *L. lactis* were grown in Elliker medium and cultured until the OD_600_ reached 0.6. Recombinant protein expression was induced with 0, 0.5, 1.0, 2.0, 4.0, or 8.0 ng/ml of nisin for 3 h, and included six biological replicates. Recombinant protein expression was further induced with 2.0 ng/ml nisin for 0, 3, 6, 9, 12, or 24 h using three biological replicates. The recombinant hsCD80 protein in the culture supernatant and pellets was detected using anti‐CD80 or anti‐Flag antibodies via ELISA and western blot, respectively (anti‐CD80 antibody, D197322, BBI Life Sciences Corporation, HK; anti‐Flag antibody, D11005, BBI Life Sciences Corporation, HK; hCD80 ELISA Kit, Boster Biological Technology Co. Ltd., Wuhan City, China).

### 4.5 Protein endocytosis analysis in vitro

Transformed *L. lactis* were grown in DMEM, and the expression of recombinant protein was induced with 2 ng/ml nisin for 6 h. The concentration of recombinant protein in the supernatant was assayed using the hCD80 ELISA Kit, and the pH value was adjusted to 7.3–7.4 with 1.0 mol/L HEPES buffer. The supernatant containing an equal amount of recombinant protein was added to the wells containing NCM460, CaCo2, HT29, SW480, and SW1116 cells for 2 h. Supernatant from the L‐vector sample was used as the negative control. Endocytosis and adsorption of recombinant protein from *L. lactis* were analyzed via immunofluorescence using a fluorescein FITC‐labeled anti‐Flag antibody.

### 4.6 Mouse splenic lymphocyte activation in vitro

BALB/c and C57BL/6 mice were euthanatized with 1% Pentobarbital Na, and splenic cells were obtained after gentle extrusion of the tissue through a 400‐mesh nylon film. Mononuclear cells were separated using lymphocyte isolate liquate (DAKEWE) according to the manufacturer's instructions, and were cultivated in the lower transwell chambers (BD Falcon) in DMEM medium supplemented with 10% FCS and 2 mM l‐glutamine. CaCo2 cells were cultivated in the upper chamber of the transwell plate. Supernatants containing recombinant proteins L‐hsCD80 and L‐CTB‐hsCD80 were added to the upper chamber to a final concentration of 1 μg/ml and incubated for 2 h. The positive control group was treated with 1 μg/ml ionomycin + 100 ng/ml PMA or 1 μg/ml anti‐PDL1 mAb. The proportion of CD3^+^CD4^+^, CD3^+^CD8^+^, and CD3^+^IFNγ^+^ T cell subsets in the lower chambers was identified via flow cytometry.

### 4.7 Protein expression and secretion in vivo

The mice were subsequently randomly classified into five groups (*n* = 30, six in each group). Mice were administered with 2 × 10^8^ recombinant *L. lactis* live bacteria intragastrically and 20 ng/ml nisin daily for 1 week. Western blotting and immunohistochemistry were used to analyze the content and distribution of the Flag label‐free hsCD80 protein in the heart, liver, spleen, kidney, stomach, intestine, intestinal contents, muscle, and fat. Analysis of the receptor‐mediated endocytosis effect of CTB was also performed.

### 4.8 Primary colon cancer immunotherapy in vivo

Male APC^min/+^ mice were fed high‐fat diets for 4 weeks to induce the development of primary colonic cancer and were subsequently randomly classified into four groups (*n* = 24, six in each group). The mice in the L‐vector, L‐hsCD80, and L‐CTB‐hsCD80 groups were treated with 0.2 ml of recombinant *L. lactis* (suspended in saline, 10^9^ CFU/ml) containing 10 ng/ml nisin by gavage every alternate day. Mice in the saline group were treated with 0.2 ml of saline by gavage every alternate day. The fecal blood, anal swelling, prolapse, and other physical conditions of the mice were observed constantly. When the control mice became very weak, all animals were sacrificed, and the tumor tissue and the heart, liver, spleen, kidney, stomach, and intestinal contents were removed completely. The size, weight, and quality inhibition rates of the tumors were measured. Each tissue was fixed using 4% paraformaldehyde solution for immunohistochemical and microscopy analysis. Cellular apoptosis was quantified using TUNEL reagent. The proportion of CD3^+^, CD3^+^CD4^+^, CD3^+^CD8^+^, and CD3^+^IFNγ^+^ T cell subsets in splenic lymphocytes was analyzed by flow cytometry. Activated CD8^+^IFNγ^+^ T cells and CD11b^+^Gr‐1^+^ MDSCs in adenoma tissues and intestinal mucosa were detected via immunofluorescence assays.

### 4.9 Melanoma lung metastases and subcutaneous xenograft immunotherapy

Female BALB/c or C57BL/6 mice (6 weeks old, *n* = 7, 35 in each group) were used to establish subcutaneous and lung metastatic cancers. The lung metastatic tumor was established via tail vein injection using 1 × 10^6^ B16F10 cells. Xenografted model mice were established by subcutaneous injection with 0.1 ml (10^7^ cells/ml) of either colon cancer CT26 or melanoma B16F10 cells in the left armpit. For melanoma lung metastases therapy, mice were treated with 0.2 ml (10^9^ CFU/ml) recombinant *L. lactis* by gavage every alternate day after tail vein injection with B16F10 cells for 3 days. For subcutaneous xenograft immunotherapy, the mice received 0.2 ml (10^9^ CFU/ml) of recombinant *L. lactis* by gavage every alternate day when the transplanted tumor reached 2 mm in diameter in 2 weeks after subcutaneous injection. Tumor‐bearing mice were randomly divided into four groups: the L‐vector, L‐hsCD80, L‐CTB‐hsCD80, and control groups. The blank control group (BC) mice were treated with 0.2 ml of saline by gavage every alternate day. When the diameter of tumors in the control group reached 2–3 cm with necrosis, all mice were sacrificed, and the tumor tissue and the heart, liver, spleen, kidney, stomach, and intestinal contents were removed completely. The size, weight, and quality inhibition rates of the tumors were measured. Each tissue was fixed with 4% paraformaldehyde solution for immunohistochemical (CD31, Ki‐67), immunofluorescence (double‐labeled CD8^+^/IFNγ^+^, double‐labeled Flag/Muc 2), and microscopy analysis. Cellular apoptosis was evaluated using TUNEL assays. The proportion of CD3^+^, CD3^+^CD4^+^, CD3^+^CD8^+^, and CD3^+^IFNγ^+^ T cell subsets of spleen lymphocytes was analyzed by flow cytometry.

### 4.10 Tumor‐specific cytotoxicity assay in vitro

To test the tumor‐specific cytotoxicity of spleen lymphocytes stimulated by recombinant protein expressed from recombinant *L. lactis*, mouse colon cancer CT‐26 cells were cultivated in 96‐well plates in triplicate using a serum‐free RPMI‐1640 medium. Subsequently, splenic lymphocytes were cocultured with CT26 colon cancer cells in a 1:20 ratio (tumor cells to lymphocytes) with 1 μg/ml CTB‐hsCD80 protein from recombinant *L. lactis* for 24 h. Cytotoxicity was evaluated by lactate dehydrogenase (LDH) release assays using an LDH Release Assay Kit (Beyotime, Cat. C0016, China). The percentage cytotoxicity was calculated as follows: LDH level in each well/total LDH released from the same number of cells lysed after two freeze–thaw cycles.

### 4.11 Active immunotherapy

CT‐26 cells (0.1 ml, 10^6^ cells/ml) were injected subcutaneously into the armpit of female BALB/c mice followed by treatment with 1 μg/ml CTB‐hsCD80 for 2 h to establish the xenograft. Saline‐treated CT‐26 cells were injected subcutaneously as the BC. When the diameter of the tumors in the control group reached 2 cm, the mice were sacrificed, and the size of the tumor was measured.

### 4.12 Immunofluorescence staining

Cells or tissue sections were fixed in 4% paraformaldehyde and methanol for 20 min at room temperature and were subsequently co‐incubated with anti‐Flag, anti‐CD8, anti‐IFNγ, anti‐MUC2, anti‐CD11b, or anti‐Gr‐1 antibodies, followed by reimaging on the BioStation (Nikon).

### 4.13 Western blotting

The expression of hsCD80 and CTB‐hsCD80 in transformed bacteria was induced using 2.0 ng/ml nisin for 6 h. The proteins in the bacterial extracts were detected by western blotting using Flag mAb (Sigma) against hsCD80 and CTB‐hsCD80, and the GAPDH mAb (MC4) (RM2002, Beijing Ray Antibody Biotech) against GAPDH as an internal control. The hsCD80 protein in the intestine and its distribution in the heart, spleen, liver, kidney, stomach, muscle, and fat were evaluated. Protein samples were separated via polyacrylamide gel electrophoresis. Next, proteins were transferred onto a polyvinylidene difluoride membrane (Millipore), which was washed with PBST, blocked in 5% nonfat dry milk, and blotted with the FLAG or GAPDH antibodies.

### 4.14 Immunocytochemistry

For Ki‐67 and CD31 staining, heat‐induced antigen retrieval was performed in 10 mmol/L citrate buffer (pH 6, Thermo Scientific), and the sections were incubated with a Ki‐67 (RD) or CD31 mAbs (RD) and stained using 3,3‐diaminobenzidine.

### 4.15 Cell staining and flow cytometry

Following the isolation of the spleen from mice, the cells were stained with fluorescently labeled antibodies CD3‐APC, CD4‐FITC, CD8a‐PC5.5, and CD69‐PE (eBioscience) for 30 min and evaluated using a CytoFlex flow cytometer (Beckman Coulter).

### 4.16 Statistical analysis

All data were expressed as the mean ± standard deviation (SD) of each group. The statistical differences between the two groups were analyzed using an unpaired Student's *t* test (two‐tailed). Multiple groups were compared using a one‐way analysis of variance (GraphPad Prism 5.0; GraphPad, Bethesda, MD). Any *p*‐values lower than 0.05 were considered statistically significant and were indicated as *p* < 0.05 (*) or *p* < 0.01 (**).

## AUTHOR CONTRIBUTIONS

Hongying Fan proposed study concepts and designed the research plan. Xiaojing Meng and Weisen Zeng gave constructive suggestions and reviewed the manuscript. Ziqin Lin, Yanqing Tang, and Zerong Chen organized the data and figures. Xueyan Xu, Xufeng Hou, Junjie Wen, and Zhenhui Chen performed the experiments and statistical analyses.

## CONFLICT OF INTEREST

The authors declare that the research was conducted in the absence of any commercial or financial relationships that could be construed as a potential conflict of interest.

### PEER REVIEW

The peer review history for this article is available at https://www.webofscience.com/api/gateway/wos/peer-review/10.1002/btm2.10533.

## TRANSLATIONAL IMPACT STATEMENT

CD80 is an important co‐stimulatory molecule that induces T cell activation and overcomes PDL1‐mediated immune suppression. However, production of recombinant sCD80 protein using traditional methods has a high production costs and inconvenient administration. We engineered a recombinant strain of *L. lactis* capable of orally delivering CTB and hsCD80 (CTB‐hsCD80), and the CTB‐hsCD80‐transformed strain having pronounced effects on the growth of subcutaneous xenografts and melanoma lung metastases. Thus, our approach could help patients inhibit tumor with low cost and high efficiency.

## Supporting information


**DATA S1:** Supporting Information

## Data Availability

The data that support the findings of this study are available from the corresponding author upon reasonable request.
