# Supplementary material for: Soluble CD80 oral delivery by recombinant Lactococcus suppresses tumor growth by enhancing antitumor immunity
Source: Bioeng Transl Med. 2023 May 3;8(4):e10533. doi: 10.1002/btm2.10533 (PMC10354755; doi:10.1002/btm2.10533)
Supplement: Supplementary file 1 — DATA S1: Supporting Information [file BTM2-8-e10533-s001.docx]

**
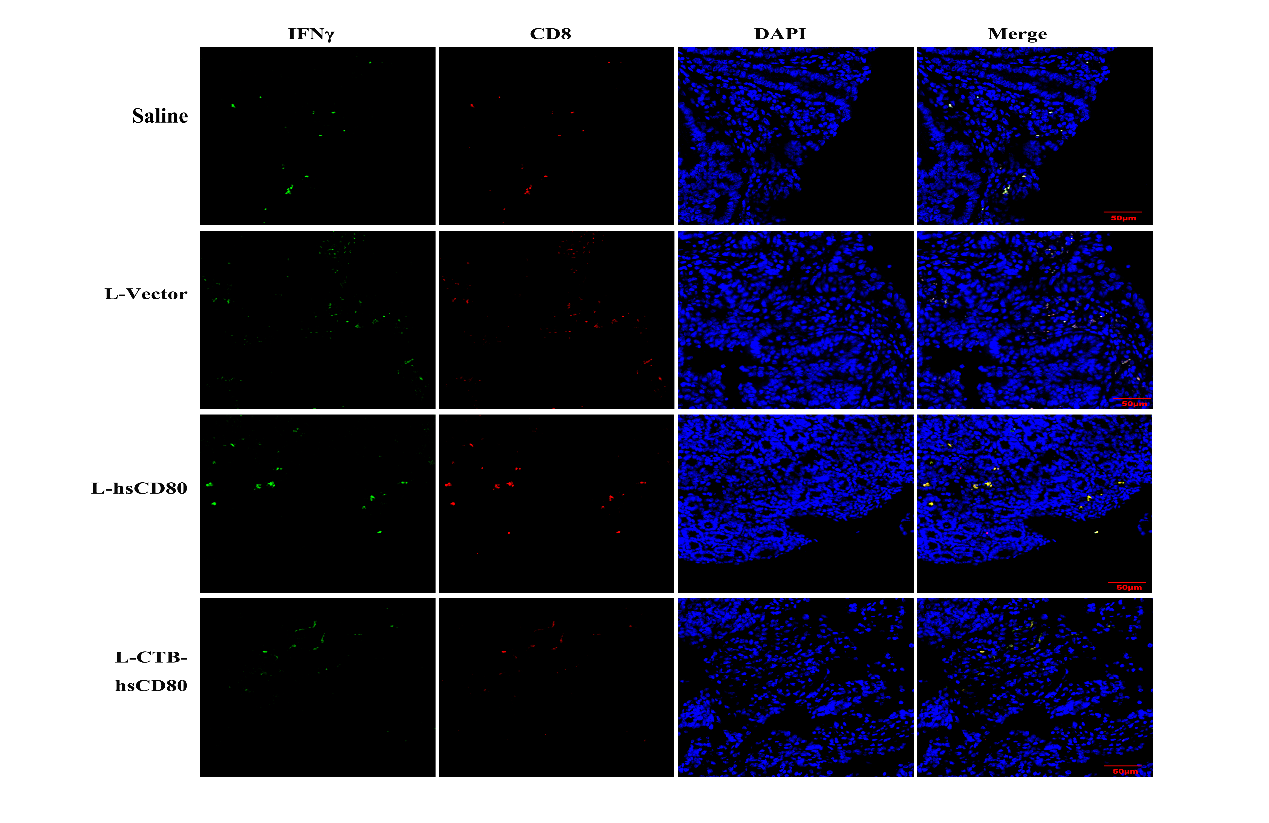
**

**Supplementary figure 1:** CD8^+^IFNγ^+^ T cells in intestinal adenoma sections imaged using immunofluorescence analysis.


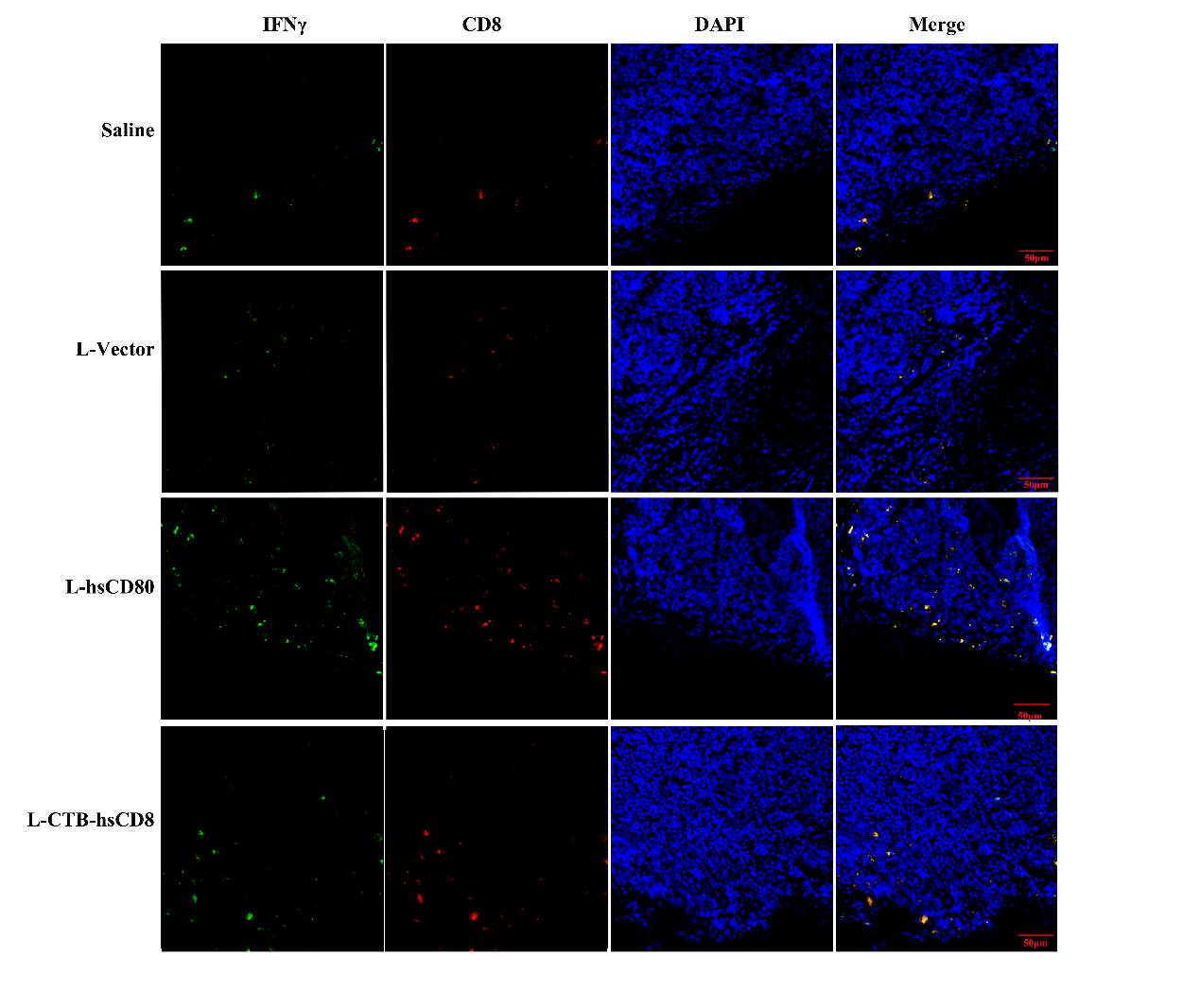


**Supplementary figure 2:** CD8^+^IFNγ^+^ T cells in sections of Peyer’s patches analyzed using immunofluorescence staining.


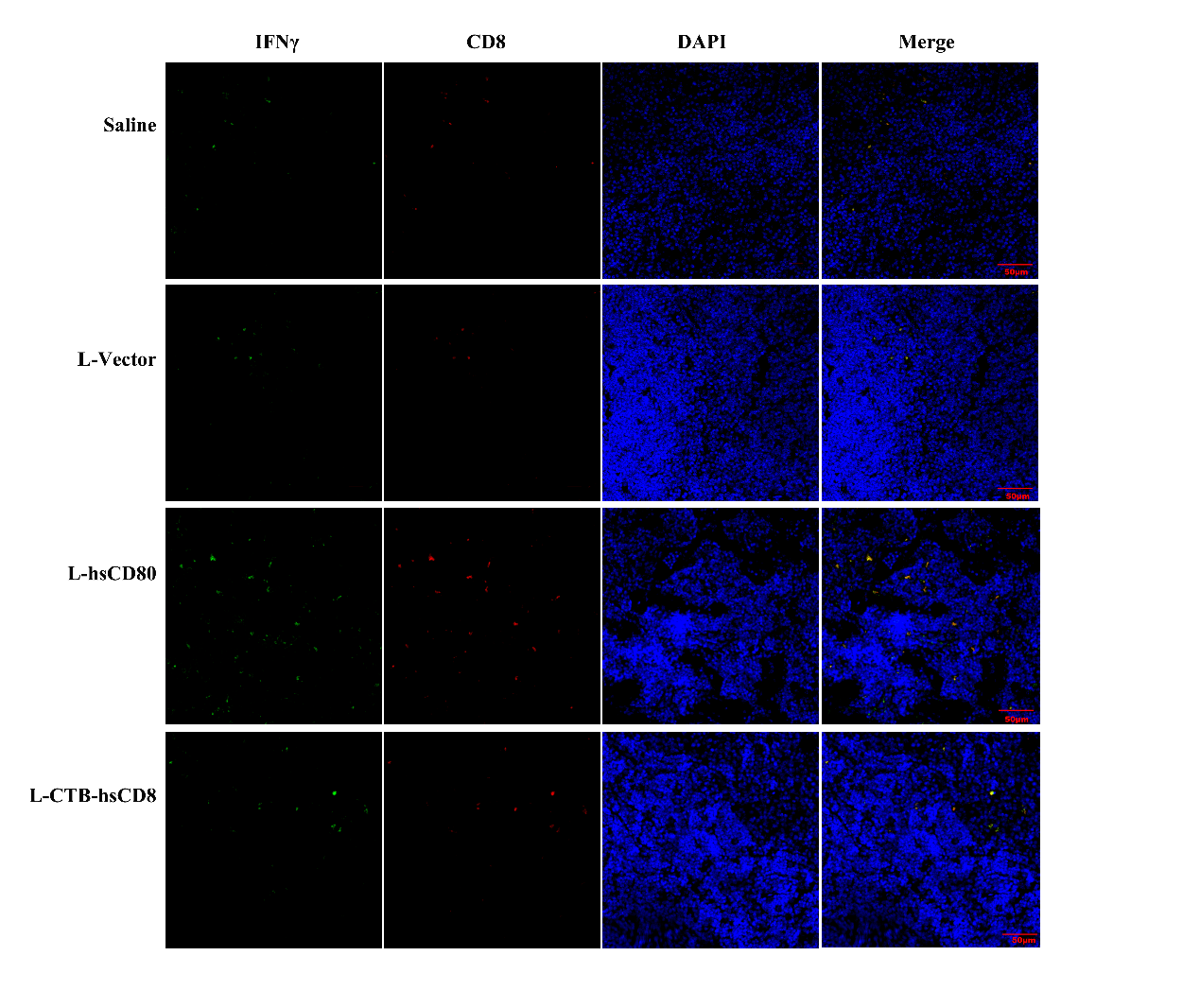


**Supplementary figure 3:** CD8^+^IFNγ^+^ T cells in mesenteric lymph node sections imaged using immunofluorescence staining.


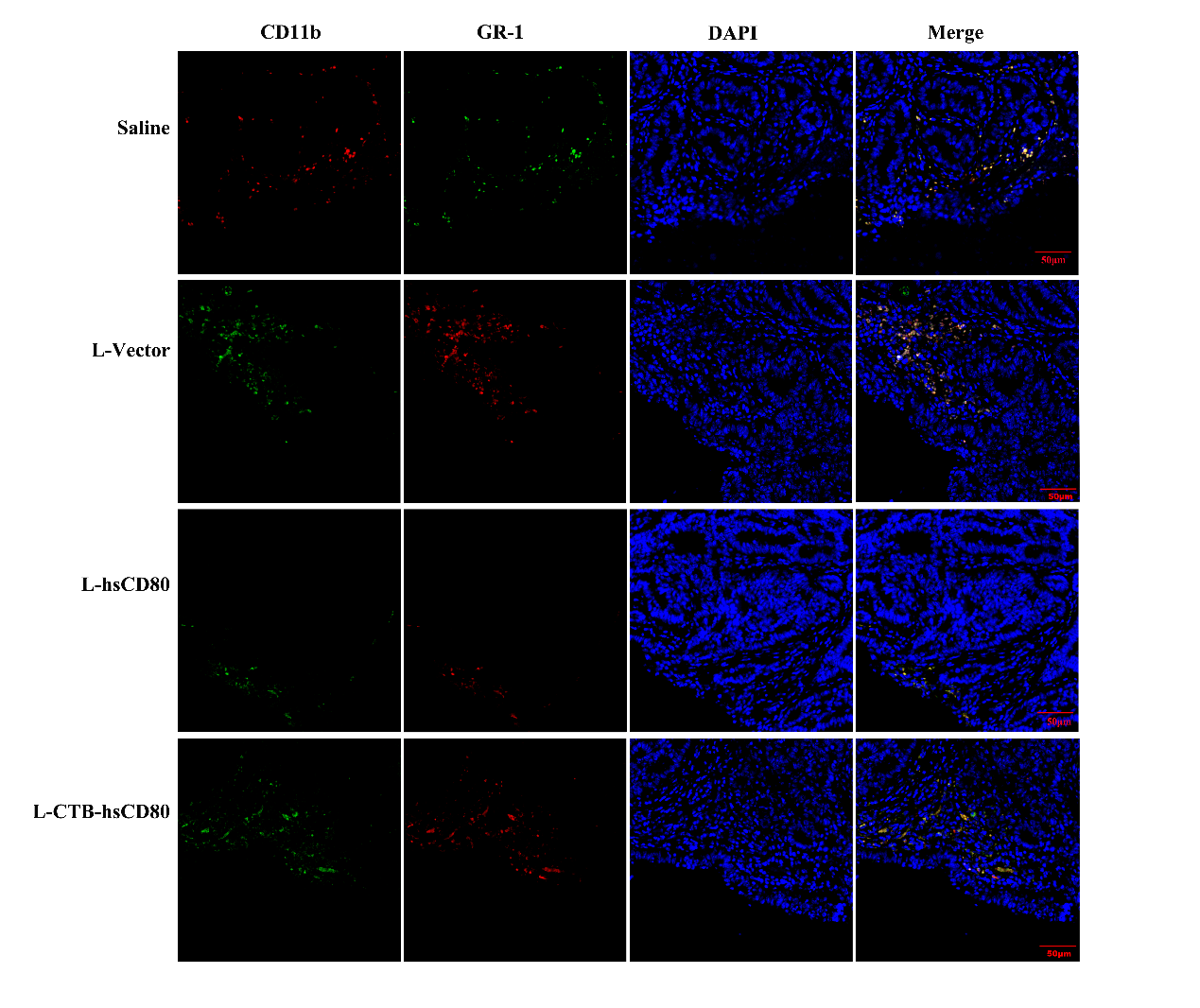


**Supplementary figure 4:** CD11b^+^Gr-1^+^ myeloid-derived suppressor cells (MDSCs) in intestinal adenoma sections imaged using immunofluorescence staining.


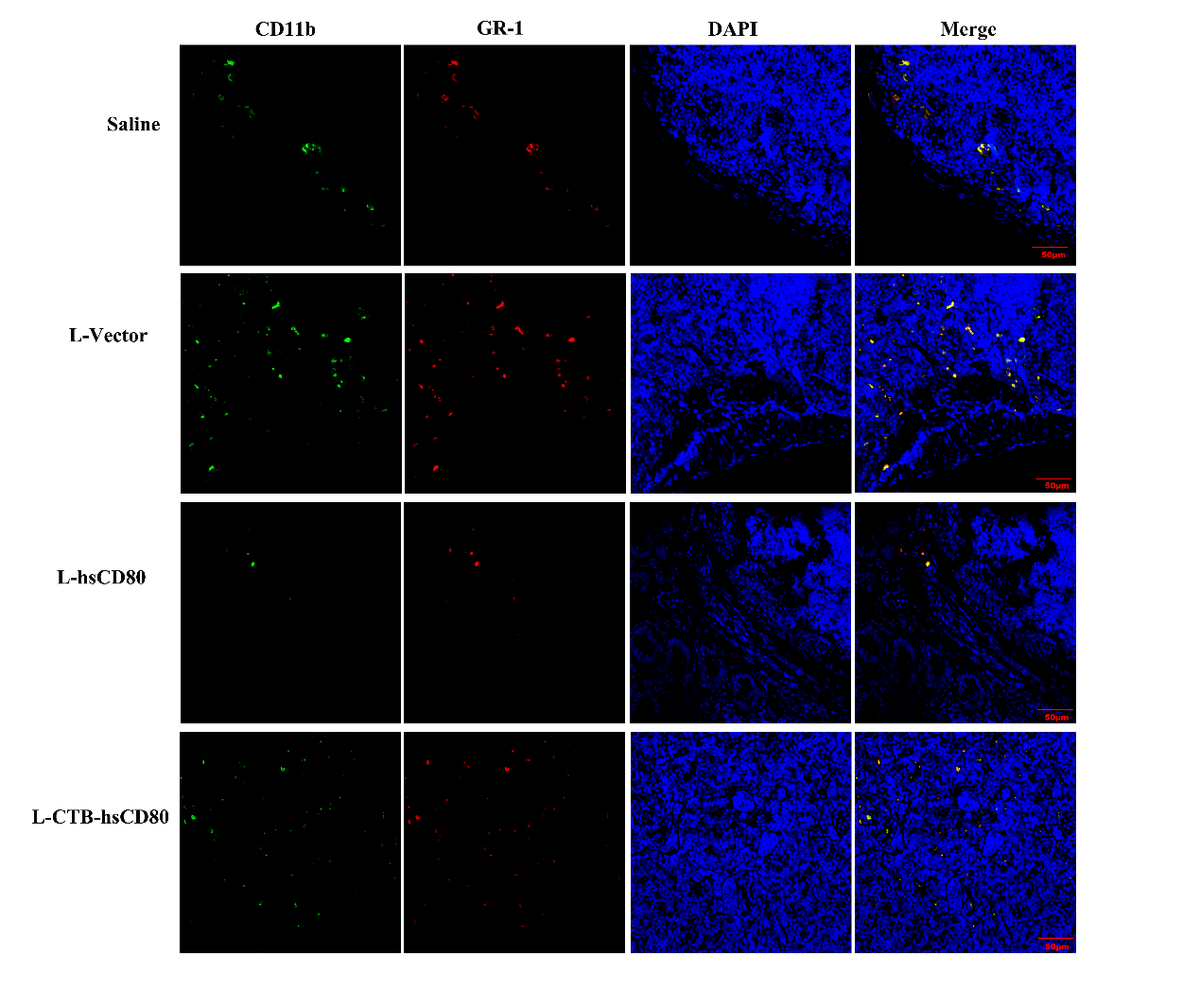


**Supplementary figure 5:** CD11b^+^Gr-1^+^ myeloid-derived suppressor cells (MDSCs) in sections of Peyer’s patches imaged by immunofluorescence staining.


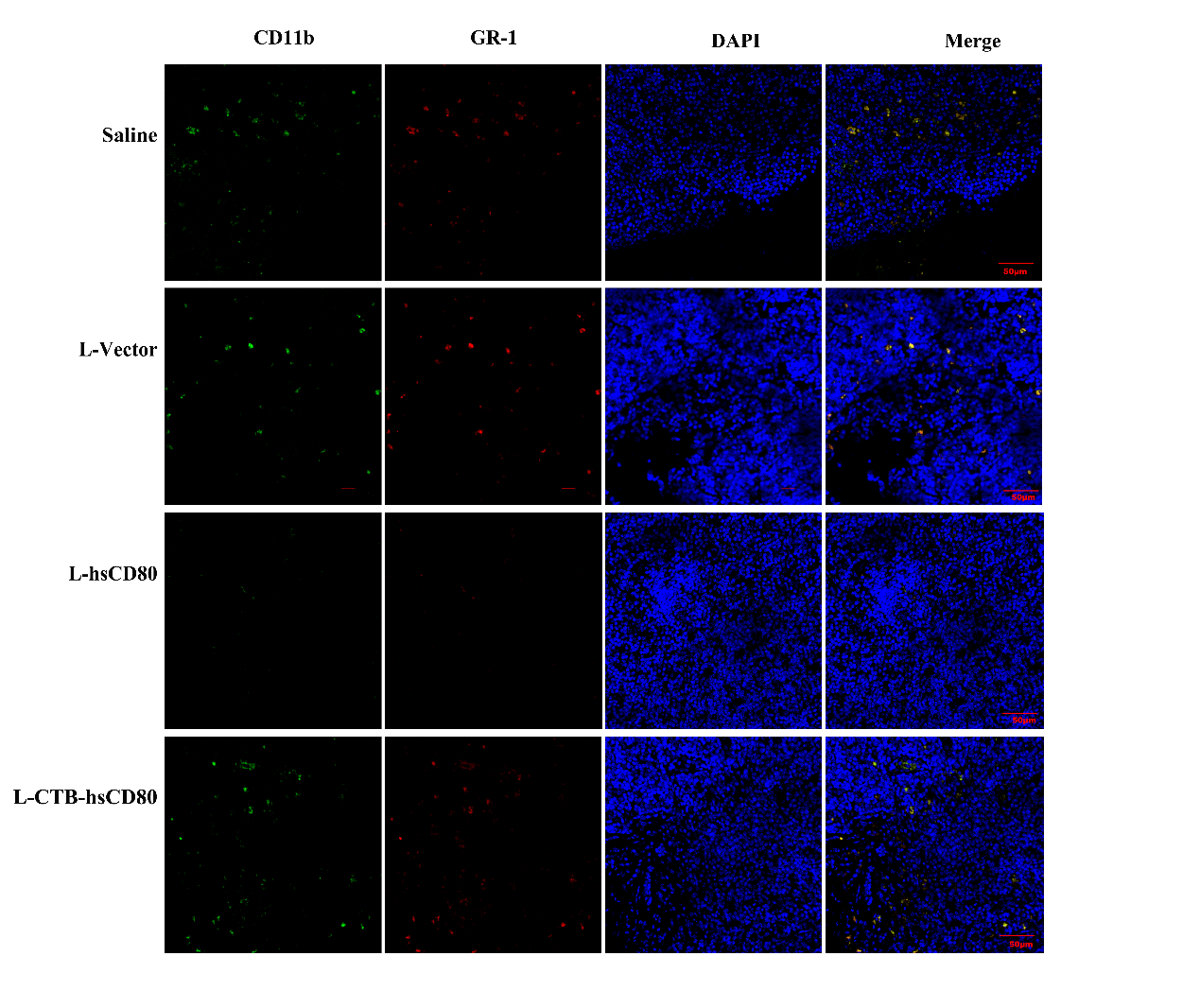


**Supplementary figure 6:** CD11b^+^Gr-1^+^ myeloid-derived suppressor cells (MDSCs) in mesenteric lymph node sections imaged by immunofluorescence staining.


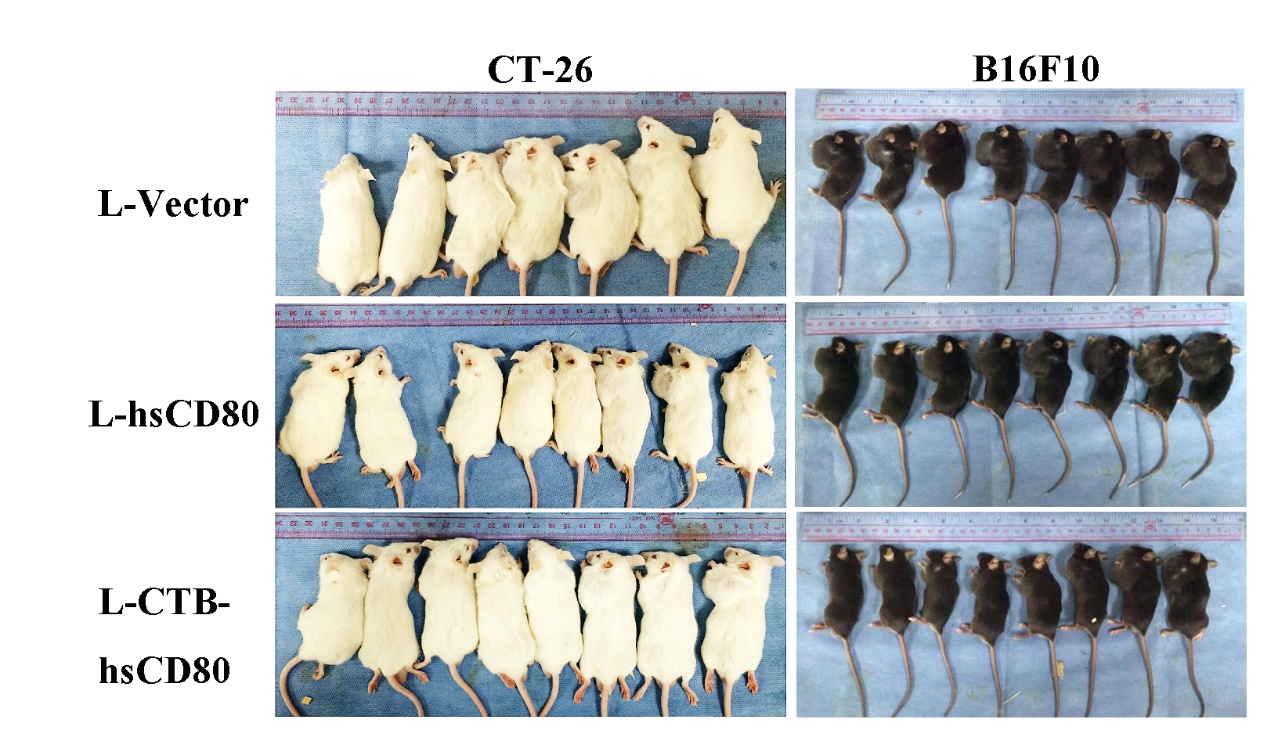


**Supplementary figure 7:** mice bearing tumors.


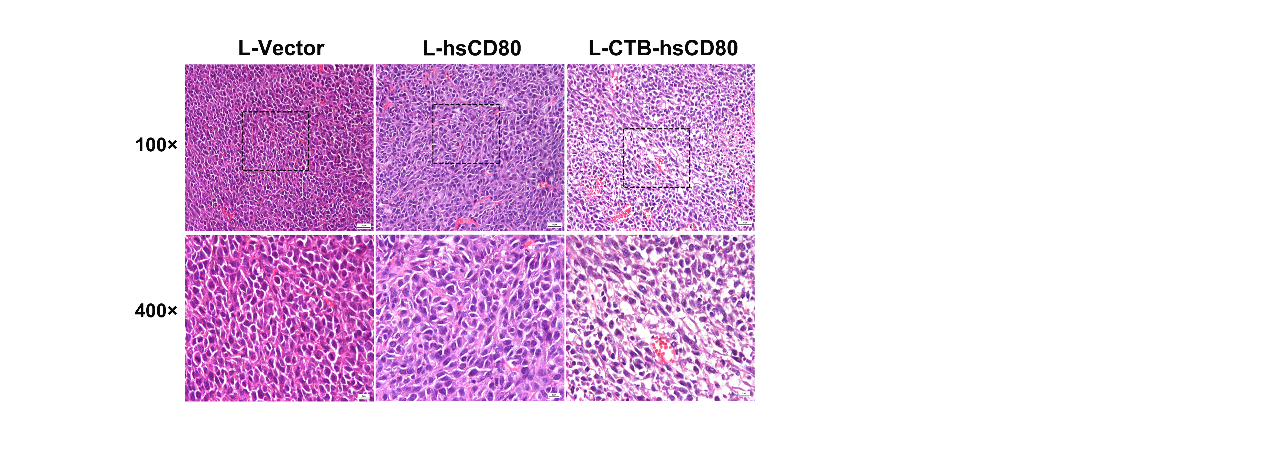


**Supplementary figure 8:** Pathological imaging of colon cancer xenografts with CT-26 strains stained with hematoxylin-eosin.


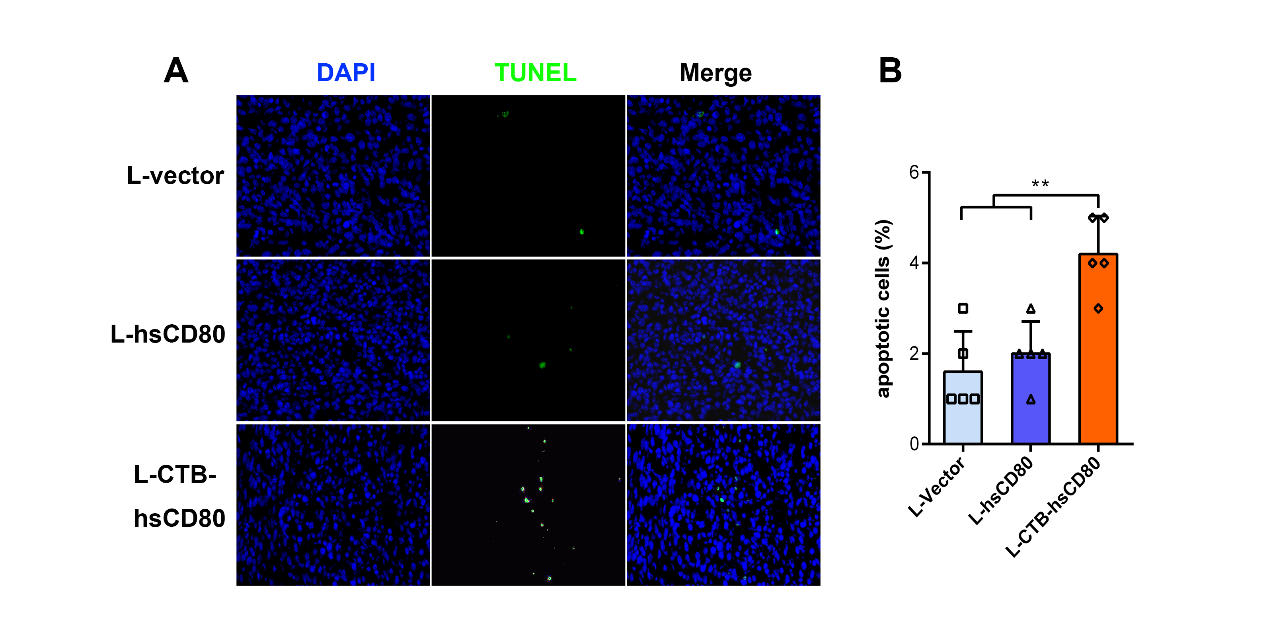


**Supplementary figure 9:** Apoptotic cells in colon cancer xenografts with the CT-26 strain imaged using TUNEL immunofluorescence staining.
